# An analysis of sugary endosperm in sorghum: Characterization of mutant phenotypes depending on alleles of the corresponding starch debranching enzyme

**DOI:** 10.3389/fpls.2023.1114935

**Published:** 2023-02-13

**Authors:** Shumpei Hashimoto, Satoshi Okada, Satoko Araki-Nakamura, Kozue Ohmae-Shinohara, Kotaro Miura, Hideo Kawaguchi, Chiaki Ogino, Shigemitsu Kasuga, Takashi Sazuka

**Affiliations:** ^1^ Bioscience and Biotechnology Center, Nagoya University, Nagoya, Japan; ^2^ Faculty of Bioscience and Biotechnology, Fukui Prefectural University, Fukui, Japan; ^3^ Engineering Biology Research Center, Kobe University, Kobe, Japan; ^4^ Department of Chemical Science and Engineering, Graduate School of Engineering, Kobe University, Kobe, Japan; ^5^ Faculty of Agriculture, Education and Research Center of Alpine Field Science, Shinshu University, Minamiminowa, Japan

**Keywords:** sorghum, sugary endosperm, debranching enzyme, starch synthesis, novel alleles

## Abstract

Sorghum is the fifth most important cereal crop. Here we performed molecular genetic analyses of the ‘SUGARY FETERITA’ (SUF) variety, which shows typical sugary endosperm traits (e.g., wrinkled seeds, accumulation of soluble sugars, and distorted starch). Positional mapping indicated that the corresponding gene was located on the long arm of chromosome 7. Within the candidate region of 3.4 Mb, a sorghum ortholog for maize *Su1* (*SbSu*) encoding a starch debranching enzyme ISA1 was found. Sequencing analysis of *SbSu* in SUF uncovered nonsynonymous single nucleotide polymorphisms (SNPs) in the coding region, containing substitutions of highly conserved amino acids. Complementation of the rice *sugary-1* (*osisa1*) mutant line with the *SbSu* gene recovered the sugary endosperm phenotype. Additionally, analyzing mutants obtained from an EMS-induced mutant panel revealed novel alleles with phenotypes showing less severe wrinkles and higher Brix scores. These results suggested that *SbSu* was the corresponding gene for the sugary endosperm. Expression profiles of starch synthesis genes during the grain-filling stage demonstrated that a loss-of-function of *SbSu* affects the expression of most starch synthesis genes and revealed the fine-tuned gene regulation in the starch synthetic pathway in sorghum. Haplotype analysis using 187 diverse accessions from a sorghum panel revealed the haplotype of SUF showing severe phenotype had not been used among the landraces and modern varieties. Thus, weak alleles (showing sweet and less severe wrinkles), such as in the abovementioned EMS-induced mutants, are more valuable for grain sorghum breeding. Our study suggests that more moderate alleles (*e.g.* produced by genome editing) should be beneficial for improving grain sorghum.

## 1 Introduction

Sorghum (*Sorghum bicolor* L. Moench) is the fifth most cultivated cereal crop after wheat, maize, rice, and barley (FAOSTAT, 2019). It is an important cereal food consumed in semi-arid regions (e.g., Africa and Asia) due to its higher drought tolerance compared to other crops, highlighting its importance for global and regional food security. Sorghum grains are rich in phenolic compounds, micronutrients, dietary fiber, and minerals (Awika and Rooney, 2004; Moraes et al., 2015; Girard and Awika, 2018; Shen et al., 2018). However, it contains inadequate levels of some essential amino acids, particularly lysine, threonine, and tryptophan (Salunkhe et al., 1977). Sorghum grains are also a safe material and are suitable for people with celiac disease, a serious condition affecting millions of individuals (Ciacci et al., 2007; de Mesa-Stonestreet et al., 2010).

Starch is the main storage form of carbohydrates in plants and its synthesis is an important process that determines yield in cereal grains. The pathway of starch synthesis involves the conversion of sucrose to ADP-glucose, which is then converted into the two types of polysaccharides, namely amylose and amylopectin. Amylopectin synthesis is highly regulated by a group of enzymes comprising ADP-glucose pyrophosphorylases (AGPases), starch synthases (SSs), starch branching enzymes (SBEs), and debranching enzymes (DBEs) (Smith et al., 1997; Myers et al., 2000; Nakamura, 2002; Ball and Morell, 2003; Pfister and Zeeman, 2016). Of these enzymes, DBEs trim the shape of amylopectin clusters by hydrolyzing α-1,6-glucosidic linkages in α-polyglucans. They are considered to play an essential role in amylopectin synthesis (Ball et al., 1996). Two types of DBEs have been identified in higher plants and green algae, namely isoamylases (ISA1, ISA2, and ISA3) and pullulanases (PUL) (Nakamura, 2015).

A mutation in the *Isa1* gene causes a dramatic change in the structure of amylopectin, whereby it becomes a randomly and more highly branched structure of *α*-1,6-/*α*-1-4-polyglucans referred to as phytoglycogen (James et al., 1995; Mouille et al., 1996; Zeeman et al., 1998; Kubo et al., 1999; Burton et al., 2002). These mutants have reduced starch content and accumulate more soluble sugars, resulting in a “sugary” endosperm phenotype. In maize, sugary endosperm has been widely used in sweet corn (*Zea mays* L.) breeding, which has a farm gate value of $1.4 billion per year in the United States (USDA-NASS, 2019). Sweet corn results from mutations in genes involved in the starch synthesis pathway, which modify the carbohydrate composition by increasing sugar content in the endosperm while reducing starch content. Several genetic loci have been shown to increase sugar content in the endosperm when mutated, including *sugary1* (*su1*), *shrunken2* (*sh2*), and *sugary enhancer1 (se1*) (Creech, 1965; Tsai and Nelson, 1966; Tracy, 1997; Rahman et al., 1998). Traditional sweet corn hybrids increased sugar content through mutations in the *su1* gene, an SBE which encodes ISA1. Compared to normal field corn, *su1* endosperms accumulate more sugar and phytoglycogen at the harvesting stage. When this enzyme is defective, the endosperm of mature kernels looks wrinkled and translucent.

Studies using *isa1* mutants and its transgenic lines in rice have revealed its central role in starch synthesis (Nakamura et al., 1997; Kubo et al., 1999; Kubo et al., 2005; Fujita et al., 2012; Shufen et al., 2019). Kawagoe et al. (2005) used a rice *isa1* mutant transformed with an amyloplast marker to track amyloplasts and starch granule formation and found that ISA1 is crucial during the early stages of starch granule formation (Kawagoe et al., 2005). Moreover, analysis of overexpression and mutant lines of ISAs revealed that ISA1 can exist as a homo-oligomer and can form a hetero-oligomer with ISA2, which is important for the synthesis of starch (Utsumi et al., 2011). As such, rice has played a central role as a model for functional studies of ISA1 in the starch synthesis pathway. However, these mutations in *ISA1* have not been exploited in rice breeding because of the severe wrinkled phenotype, compared to maize, for which weak mutants were obtained.

Literature on the history of sugary sorghum may go back over half a century (Karper and Quinby, 1963). There, it was reported that sugary seeds contain at least twice as much total sugar as normal seeds. Moreover, the people of India have undoubtedly known of the peculiar sweetness of sugary endosperms for a long time. This sugary phenotype was assumed to be caused by a single recessive gene, and the corresponding gene was designated *Su* (Karper and Quinby, 1963). However, an analysis of the corresponding gene has not been conducted and the molecular mechanism of the sugary trait remains unknown. In this study, we studied a variety of sugary sorghum, namely ‘SUGARY FETERITA’, which shows the typical sugary endosperm phenotype. Molecular genetic studies revealed that the corresponding gene, *Su*, encodes sorghum ISA1 (SbISA1).

## 2 Materials and methods

### 2.1 Plant materials

The sorghum (*Sorghum bicolor* L. Moench) accessions ‘SUGARY FETERITA’ (SUF, JP No. 44623) and ‘FETERITA FAYOUMI D.S.21’ (FET, JP No. 246696) were obtained from Genebank Project, NARO (National Agriculture and Food Research Organization) and used for the phenotypic and genetic characterization of the sugary endosperm and gene expression. ‘Nakei MS-3B’ (MS3B) and SUF were used for segregation analysis and rough mapping. For complementation analysis using rice, the *sugary-1* mutant SG0807 was obtained from Oryzabase (https://shigen.nig.ac.jp/rice/oryzabase/). Sorghum ethyl methanesulfonate (EMS) mutants (PI 678144, PI 678119, and PI 678151) were obtained from the Functional Gene Discovery Platform for Sorghum (https://www.purdue.edu/sorghumgenomics). For haplotype analysis, the Nagoya University Panel (NUP; 187 sorghum accessions) was obtained from the sorghum diversity research set of NARO (National Agriculture and Food Research Organization) (Shehzad et al., 2009), the US historic sweet sorghum collection (Wang et al., 2009), and the available lines in our lab, were used for this study (**Supplementary Table S2**).

### 2.2 Rough mapping of the sugary locus in SUF

For rough mapping, seeds exhibiting a wrinkled trait in the F_2_ progeny, resulting from a cross between MS3B and SUF, were used. Genomic DNA was isolated from ca. 2-week-old leaves from 96 individuals using the cetyltrimethylammonium bromide (CTAB) extraction method (Murray and Thompson, 1980) with some modifications. Briefly, leaf samples were ground using a MultiBead Shocker (Yasui Kikai, Osaka, Japan) in 2 × CTAB extraction buffer (100 mM Tris-HCl [pH 8.0], 50 mM EDTA, 1.4 M NaCl, 2% [w/v] CTAB, 1% [w/v] PVP). After incubating at 60°C for 30 min, an equal volume of chloroform was added. After centrifugation at 15,000 rpm for 5 min, the supernatant was recovered, and an equal volume of isopropyl alcohol was added. The sample was recovered by centrifugation at 15,000 rpm for 5 min and the pellet was washed with 70% (v/v) ethanol. The DNA pellet was dried and dissolved in 1 × Tris/EDTA (TE) solution (10 mM Tris-HCl [pH 8.0], 1 mM EDTA [pH 8.0]). The purified DNA samples were then used for genotyping using a polymerase chain reaction (PCR). Simple sequence repeat (SSR) markers, as reported by Yonemaru et al. (Yonemaru et al., 2009), were screened and 178 markers showing polymorphisms between MS3B and SUF were selected (**Supplementary Table S3**). DNA segments were amplified by PCR using the following program: 95°C for 1 min; 30 cycles of 95°C for 30 sec, 55°C for 30 sec, and 72°C for 30 sec; 72°C for 7 min. The PCR products were analyzed by agarose gel electrophoresis.

### 2.3 Gene expression analysis

‘SUGARY FETERITA’ and ‘FETERITA FAYOUMI D.S.21’ were grown in the greenhouse and immature seeds were sampled 0, 7, 14, and 21 days after flowering (DAF). These samples were immediately frozen in liquid nitrogen and crushed with a mortar and pestle until a powder was obtained. Next, RNA extraction buffer (50 mM Tris-HCl [pH 9.0], 20 mM EDTA [pH 8.0], 200 mM NaCl, 1% (w/v) Sodium N-lauroyl sarcosinate, and 5 mM DTT) was added to each sample and centrifuged at 14,000 rpm for 5 min at 4 °C. The supernatant was used in the subsequent steps. Total RNA was isolated from each sample using the TRIzol^®^ reagent (Invitrogen, Carlsbad, CA, USA) according to the manufacturer’s instructions. Next, 500 ng of total RNA was used to synthesize first-strand cDNA using an Omniscript reverse transcription (RT) kit (Qiagen, Hilden, Japan), according to the manufacturer’s instructions. Quantitative RT-PCR (qRT-PCR) was conducted using the KOD SYBR^®^ qPCR Mix (TOYOBO, Osaka, Japan) and a real-time thermal cycler (Bio-Rad Laboratories, Hercules, CA, USA). The ubiquitin (*UBQ*) gene served as the internal reference for the qRT-PCR analyses. Primers used in the analysis were listed in **Supplementary Table S3**.

### 2.4 Phenotypic analysis of grains

We examined the morphological traits of sorghum kernels using a stereo microscope. Seeds were cut with a razor blade and cross-sections were observed using the same microscope. For histological analysis, seed samples were fixed in FAA fixative (formaldehyde and acetic acid in 50% [v/v] ethanol) under a vacuum and dehydrated in a graded ethanol series. Samples were embedded in Technovit 7100 (Kulzer, Hanau, Germany) and sectioned at a thickness of 1 μm with a manually operated rotary microtome (Leica RM2125RT). Sections were stained with iodine solution (1% [w/v] KI, 0.1% [w/v] I_2_) and observed under a light microscope (Olympus BX5). Photographs were taken using a digital camera (Olympus DP74) connected to the microscope. Brix values of the seeds were obtained as follows; first, 0.2 g of seeds were roughly crushed with a hammer and placed in a tube with beads. They were then crushed in a grinding apparatus (Bio-Medical Science Co., Ltd, BMS-A20TP) for at least 20 min at 1,100 rpm. To the powdered sample, 200 µL of water was added and mixed in the grinding device for a further 5 min at 1,100 rpm. Finally, the sample was centrifuged at 15,000 rpm for 5 min and the supernatant was measured with a refractometer (AS ONE, APAL-1).

### 2.5 Agrobacterium-mediated rice transformation

Genomic DNA fragments (8.6 kb) containing Sobic.007G204600 from MS3B and SUF were introduced into pCAMBIA1300 (Cambia) using the KpnI/EcoRI sites. Each construct was transformed into *Agrobacterium tumefaciens* EHA105 using an electroporator (Eppendorf) at 2,500 V and used to infect calli of *sugary-1* mutant (SG0807), whose genetic background is *Oryza sativa* (L.) cv. ‘Kinmaze’ as described by Ozawa (Ozawa, 2012). Transformed calli and plants were selected by hygromycin resistance, with regenerants grown in pots in a greenhouse.

### 2.6 Haplotype analysis

All accessions were sequenced using an Illumina Hiseq X Ten sequencer (Illumina, San Diego, CA, USA) by pair-end sequencing to obtain SNPs as previously reported (Hashimoto et al., 2021). For SNP calling, clean reads were aligned with the BTx623 (v3.0.1) reference genome using Burrows-Wheeler Alignment (BWA, v0.7.17) (Li and Durbin, 2009) with default parameters. Variants were called independently and merged using the Genome Analysis Toolkit (GATK, v4.1.8, HaplotypeCaller) (McKenna et al., 2010). After the genomic region of *SbSu* (Sobic.007G204600) was extracted using VCFtools v0.1.16 (Danecek et al., 2011) from the resulting merged VCF file, haplotypes were categorized based on nonsynonymous SNPs associated with single amino acid substitutions in the coding region.

## 3 Results

### 3.1 Phenotypic characterization of ‘SUGARY FETERITA’

**Figure 1:**
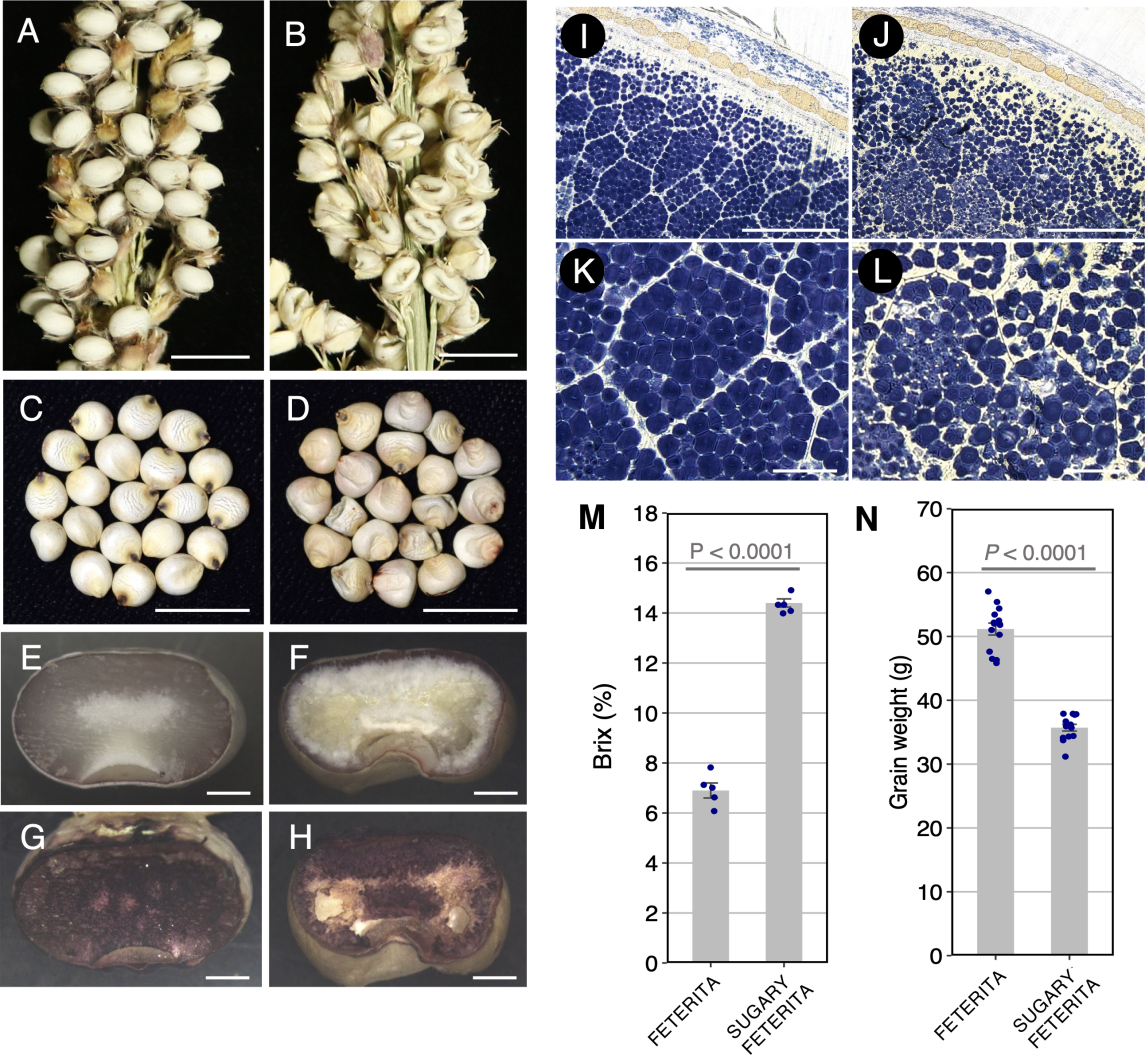
Seed phenotypes of the two varieties, ‘FETERITA’ (FET) and ‘SUGARY FETERITA’ (SUF). **(A, D)** Panicles of FET **(A)** and SUF **(B)**. Kernels of FET (normal, **C**) and SUF (wrinkled, **D**). **E–G**, Transverse sections of FET and SUF stained with iodine solution **(G, H)**. **(K–N)**, Cross sections of FET **(I, K)** and SUF **(J, L)**. Aberrant starch structures were observed in **(L)**. **(M, N)** Brix values **(M)** and grain weights **(N)** of the seeds. Scale bars: 1 cm in **(A–D)**, 1 mm in **(E–H)**, 200 μm in **(I, J)**, and 50 μm **(K, L)**.

During the vegetative and heading stages, SUF showed no obvious differences from the wild type FETERITA (FETERITA FAYOUMI D.S.21; FET) (**Supplementary Figure S1**). However, at the hard dough stage, grains of SUF exhibited wrinkled and translucent phenotypes whereas FET had normal, smooth grains (**Figures 1A–D**). Grains stained with iodine solution revealed that the endosperm of FET stained well in dark blue (**Figures 1E, G**), while that of SUF stained less overall, with no staining in the central region (**Figure 1F, H**). The detailed observation of the cross-sections revealed that SUF had loosely packed and abnormal starch granules compared to the densely packed granules of FET (**Figures 1I-L**). The Brix value of SUF was 2 fold higher than FET (**Figure 1M**). A decrease of grain weight was also observed in SUF (**Figure 1N**). These results indicate that the starch components of the endosperm of SUF differ from FET, resulting in the wrinkled grain phenotype and the accumulation of soluble sugars.

### 3.2 Positional mapping and identification of the corresponding gene

It is known that sugary mutants of maize and rice present wrinkled seeds, therefore, we used this phenotype as a morphological indicator of sugary endosperm in sorghum. For positional mapping, a Japanese sorghum variety, MS3B (a maintainer line with a normal phenotype), and SUF were crossed. The resulting MS3B x SUF (F_1_) seeds showed a normal phenotype (**Supplementary Figure S2**), suggesting that the sugary endosperm phenotype is recessive. We then analyzed the segregation pattern of the 1,627 F_2_ seeds and found 1,212 were wild-type seeds and 415 seeds with the wrinkled phenotype. The segregation ratio of normal to wrinkled seeds in the population fit to 3:1 *via* the chi-square test (**Supplementary Table S1**), suggesting that the seed wrinkling trait is a recessive, single-gene trait.

**Figure 2:**
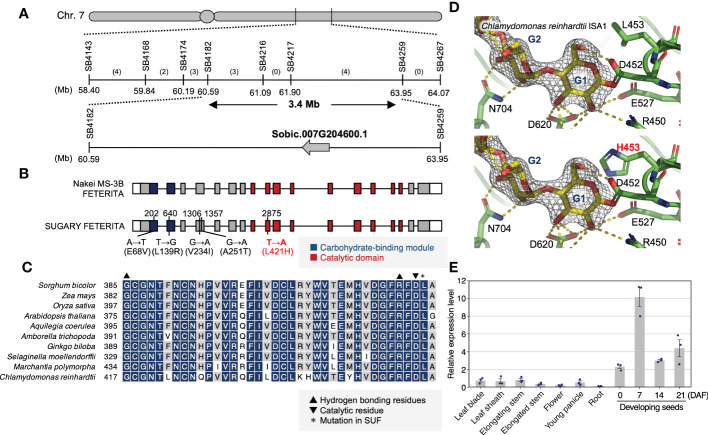
Positional cloning of the corresponding gene for sugary endosperm in ‘SUGARY FETERITA’. **(A)** A high-resolution map of the candidate locus on chromosome 7. Vertical lines indicate the positions of the SSR markers with their physical positions (Mb) in Phytozome 13 (*Sorghum bicolor* v3.1.1). The number of recombinants is shown between the markers. The mapping analysis narrowed the candidate region to a 3.4 Mb stretch between SB4182 and SB4259 on the long arm of chromosome 7. Around 400 genes were annotated in this region, including Sobic.007G204600. **(B)** Gene structures of *SbSu* according to the three cultivars, ‘Nakei MS-3B’(MS3B), ‘FETERITA’(FET), and ‘SUGARY FETERITA’(SUF). Exons are represented as boxes and introns as the lines between boxes. White, grey, black, and red boxes indicate the 3’- or 5’-untranslated regions (UTR), protein-coding regions, a carbohydrate-binding module, and a catalytic domain, respectively. SUF has five SNPs in its coding region compared to the MS3B and FET alleles. **(C)** The conservation of L421 among plants and algae is indicated with an asterisk. It is located in the catalytic domain, indicated by an inverted triangle. (**D**, top) The expanded structure of *Chlamydomonas reinhardtii* Isoamylase 1 (CrISA1) obtained from PDBj (Protein Data Bank Japan, PDB DOI: 10.2210/pdb4J7R/pdb) and visualized in PyMOL v2.1. The L453 in *C reinhardtii*, which corresponds to L421 in FET, was substituted to Histidine (bottom, highlighted in red). **(E)** Expression pattern of *SbSu* among organs. Error bars indicate the standard error of three biological replicates.

Positional mapping using 96 F_2_ wrinkled phenotype seeds revealed genomic regions on chromosome 7 (60.6–65.0 Mb) and 9 (55.1–59.2 Mb) with biased segregation ratios (**Supplementary Figure S3**). Although the bias on the latter region did not fit the segregation pattern described above (the recessive mutation model), the former biased for the SUF homozygotes fit it (**Supplementary Table S1**). In the region on chromosome 7, we searched candidate genes (i.e., starch synthesis-related genes). Here, we found Sobic.007G204600, which encodes a DBE (SbISA1), localized within 3.4 Mb on the candidate region (**Figure 2A**). It should be noted that Sobic.007G204600 is the sorghum homolog of maize *Su1* (**Figure 2A**). Sequencing analysis of the genomic DNA of the Sobic.007G204600 of MS3B and FET revealed that these genes produce proteins with the same amino acid sequences as BTx623, the reference genome (**Figure 2B**). Conversely, the allele in SUF had five SNPs which resulted in nonsynonymous amino acid substitutions in the protein-coding region (**Figure 2B**). One of those in SUF (L421) was next to the highly conserved catalytic residue (D420) of ISA1 (**Figure 2C**) (Sim et al., 2014). A protein structure analysis using *Chlamydomonas reinhardtii* ISA1 (CrISA1) suggested that L453H (corresponding to L421H in SUF) could sterically hinder the binding of the catalytic residue (**Figure 2D**). Gene expression analysis by qRT-PCR indicated that Sobic.007G204600 was highly expressed in developing seeds (**Figure 2E**). From these results, we supposed that this gene might play a role in starch metabolism in endosperm cells and that mutations therein result in their loss of function. Therefore, it is a candidate for the corresponding gene of SUF.

### 3.3 Complementation of the rice *sugary-1* mutant

**Figure 3:**
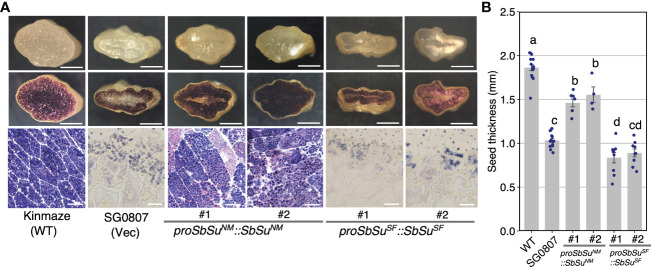
Complementation of the rice *su1* mutant with *SbSu*. A DNA fragment containing a 2 kb promoter fragment followed by the *SbSu* coding region (NM; Nakei MS-3B, SF; SUGARY FETERITA) was introduced into the rice *su1* mutant, SG0807. The SG0807 mutant (vec) and Kinmaze (wild-type) plants transformed with an empty vector were used as controls. **(A)** Grain morphologies of wild-type rice (Kinmaze), SG0807, and transgenic seeds. Crossed sections shown in the upper pictures were stained with iodine solution (middle) to observe starch structures (bottom). Scale bars: 1 mm (upper and middle) and 20 μm (bottom). **(B)** Comparison of seed thickness in wild-type, SG0807, and transgenic lines. For statistical analysis, an ANOVA model was followed using Tukey’s HSD test to estimate statistical differences. Different lowercase letters in each plot indicated significant differences in the *post-hoc* test (*P* < 0.05).

To confirm that the SUF allele of Sobic.007G204600 is a loss-of-function allele, we performed a complementation test using the rice *sugary-1* (*osisa1*) mutant line (SG0807), which exhibits thin seeds with distorted starch, resulting in a less stained endosperm following iodine staining (**Figure 3**). A genomic DNA fragment containing the promoter (2 kb upstream from ATG) and the coding region from MS3B or SUF was introduced into SG0807. The results showed that the endosperm of transgenic lines transformed with coding region of MS3B stained positive with iodine (**Figure 3**) and almost recovered the seed thickness of SG0807. In contrast, the endosperm of transgenic lines complemented with the genomic fragment derived from SUF did not stain the same level as SG0807 (**Figure 3**). These results suggested that the corresponding gene is Sobic.007G204600 (*SbSu*), and that the haplotype of SUF (pronounces E68V, L139R, V234I, A251T and L421H mutations) causes sugary endosperm, and the MS3B and FET alleles of this gene are gain-of-function alleles, whereas SUF allele is a loss-of-function allele. This sugary gene *Su*/*su* assigned firstly by Karper and Quinby (1963) was redefined as *SbSu* in the later sections.

### 3.4 Expression profiles of starch synthesis genes

To understand how the mutation (L421H) in SUF affects starch synthesis, we examined the expression of 16 starch synthesis-related genes (**Supplementary Figure S4**) using qRT-PCR. These genes include four DBEs (*SbSu*, *SbIsa2*, *SbIsa3*, and *SbPul1*) and four SBEs (*SbSbeI*, *SbSbeIIa*, *SbSbeIIb*, and *SbSbeIII*). The DBEs catalyze the hydrolysis of α-1,6-glucosidic linkages in α-polyglucans, while SBEs catalyze the formation of α-1,6-glucosidic linkages in amylopectin and glycogen. The other starch synthesis-related genes include an SS (*SbSsI*), four transporters (*SbSweet2-1*, *SbSweet4-3*, *SbBt1*, *SbPho1*), an AGPase (*SbSh2*), and an ortholog of *se1* in maize (*SbSe1*).

**Figure 4:**
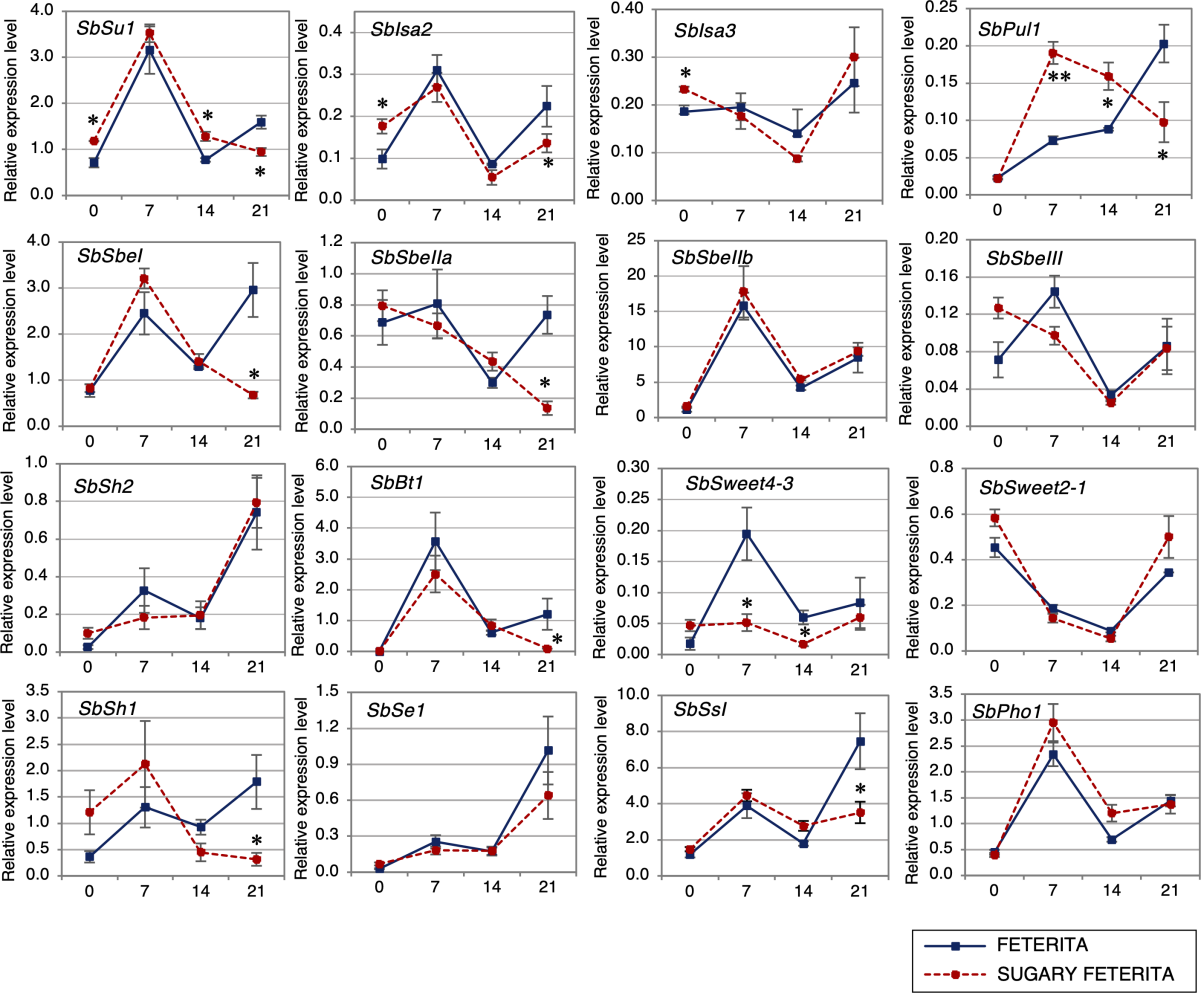
Expression profiles of starch synthesis genes of sorghum during seed development in wild-type ‘FETERITA’ and ‘SUGARY FETERITA’. Total RNA was extracted from seeds at 0, 7, 14, and 21 days after flowering (DAF), and qRT-PCR was conducted for 16 genes; *SbSu* (Sobic.007G204600), *SbIsa2* (Sobic.009G127500), *SbIsa3* (Sobic.002G233600), *SbPul1* (Sobic.006G015800), *SbSbe1* (Sobic.010G273800), *SbSbeIIa* (Sobic.006G066800), *SbSbeIIb* (Sobic.004G163700), *SbSbeIII* (Sobic.003G213800), *SbSh2* (Sobic.003G230500), *SbBt1* (Sobic.004G085100), *SbSweet4-3* (Sobic.004G133600), *SbSweet2-1* (Sobic.001G377600), *SbSh1* (Sobic.010G072300), *SbSe1* (Sobic.005G199300), *SbSsI* (Sobic.010G047700), *SbPhoI* (Sobic.001G083900). Expression levels are shown relative to the expression of the sorghum ubiquitin (*SbUbi*; Sobic.001G311100) gene. All data are shown as means ± SE from three biological replicates. Two-tailed unpaired t-tests were used to determine significant differences. *P < 0.05 and **P < 0.01. More detail about each target gene can be found in **Supplementary Figure S4**.

At the early seedling stage (ca. 14 DAF), the expression levels of the two DBEs (*SbSu1* and *SbPul*) were upregulated in SUF, indicating positive feedback due to the loss-of-function of *SbSu1* (**Figure 4**), although th other DBEs (*Sbisa2* and *Sbisa3*) were not upregulated. During the late stage (21 DAF), the expression of *SbSbeI*, *SbSbeIIa*, *SbSsI*, *SbSe1*, *SbBt1*, *SbSh1*, and three DBEs (*SbSu1*, *SbIsa2* and *SbPul1*) was significantly downregulated in SUF (**Figure 4**). The expression of *SbSweet4-3* was markedly lower in SUF at 7 and 14 DAF. There were no significant changes for *SbSbeIIb* and *SbSh2* (**Figure 4**). These observations suggest that a fine-tuning activity works for the appropriate expression of DBEs during the early stage, while during the later stage, the activity disappears in the starch synthesis pathway.

### 3.5 Haplotype analysis of the *SbSu* gene

**Figure 5:**
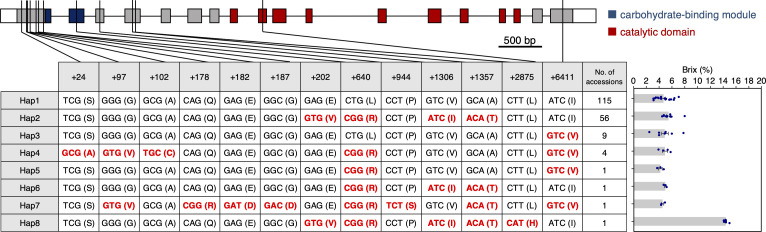
Haplotype analysis of *SbSu*. Eight haplotypes were observed in 187 accessions based on nonsynonymous SNPs in the coding region. Schematic diagram of *SbSu* is shown on the top (also, see **Figure 2B**). The number in the first row of the table indicates the relative physical position from the transcription start site. Nonsynonymous mutations are indicated in red. For Hap5-7 (only one accession), data were obtained from more than three biological replicates.

If *SbSu* is used for modern breeding in grain sorghum, it is estimated that weak alleles were widely used during the establishment of those landraces and varieties. To find weak allels, we did a haplotype analysis using a NUP containing 187 accessions (see Materials Methods). Here, 13 nonsynonymous SNPs were found in the coding region of *SbSu*, which resulted in 8 haplotypes (**Figure 5**). Interestingly, the accession containing Hap 8 is the only SUF among the NUP accessions. Subsequently, we measured the Brix values for each haplotype and found that the values were mostly similar except for Hap 8 (SUF), which had a higher score than the rest (**Figure 5**). These results suggest that although Hap 8 (SUF allele) shows a high Brix value, it is not used or spread in NUP. It is supposed that the sugary endosperm of Hap 8 is too strong of a phenotype, decreasing grain weight and wrinkled seed, thereby decreasing the germination rate. It should be noted that the five amino acid substitutions (E68V, L139R, V234I, A251T and L421H) observed in Hap 8 (**Figure 2B**) compared with Hap 1; however, four non-synonymous substitutions (E68V, L139R, V234I, and A251T) were the same as in Hap 2. Considerd that the Brix score of Hap 8 was high, although that of Hap 2 was almost as low as that of Hap 1, the L421H substitution (caused by the SNP of T2875A) should be the corresponding mutation for the sugary phenotype of SUF.

### 3.6 Identifying a novel weak allele of *SbSu*

**Figure 6:**
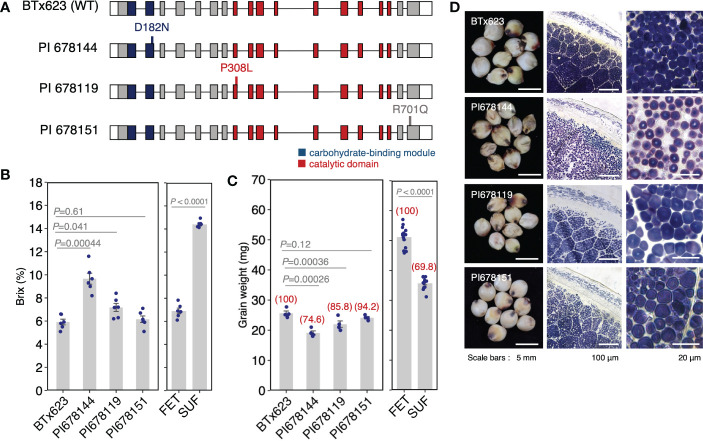
Novel sugary mutants of sorghum. **(A)** The position of the mutation in *SbSu* for each mutant. **(B**, **C)** Comparison of Brix values **(C)** and grain weight **(D)** of the mutants. In panel **(C)** red numbers on each bar with parentheses indicate the relative ratio of grain weight against wild-type. Data for FET and SUF are the same as in **Figure 1 M, N**. **(D)** Cross sections of seeds of *SbSu* mutants induced by EMS (see Materials and Methods).

The previous results reveal the importance of a weak allele for breeding; i.e., showing both a relatively high Brix value and a relatively normal seed shape (**Supplementary Figure S5**). However, we could not find a weak allele in the NUP accessions. Therefore, we searched the sorghum mutant panel of the Functional Gene Discovery Platform for sorghum (https://www.purdue.edu/sorghumgenomics). The accessions in the platform were mutagenized by EMS, generating 12,000 mutant families in the BTx623. Genome sequences were generated for 586 EMS lines and bioinformatic processing was used to identify all mutations that impact predicted protein sequences. We searched the mutants of Sobic.007G204600 and found three mutants: PI 678144, PI 678119, and PI 678151. The mutations comprised single nonsynonymous amino acid substitutions; D182N (PI678144), P308L (PI678119), and R701Q (PI678151) (**Figure 6A**). It should be noted that these mutations were located in the highly conserved region (**Supplementary Figure S6**), and especially, D182 and P308 were located in the catalytic domain and carbohydrate-binding module, respectively. To study how these mutations influence endosperm phenotype, the Brix value and grain weight were analyzed and compared to BTx623 (wild-type). Of the three mutants, PI 678144 is an intermediate phenotype of sugary endosperm, with a Brix value of 9.6 compared with 5.9 in wild-type and 14.4 in SUF (**Figure 6B**). For grain weight, the relative value of PI 678144 against wild-type was 74.6, which was higher than that of SUF (69.8) (**Figure 6C**). It was supported by the section stained with an iodine solution of PI 678144, which showed small and distorted starch granules, but seemed a weaker phenotype than that of SUF (**Figure 6D**). The Brix value of PI678119 was slightly higher than that of the wild-type, and the sections also indicated that it had a weaker phenotype than that of PI 678144. The phenotype of PI678151 was similar to the wild-type (**Figure 6B-D**). These results suggested that PI678144 (D182N) is an appropriate weak allele, which could make it valuable for the breeding of sugary endosperm.

## 4 Discussion

In 1963, Karper and Quinby stated the following: “A sugary endosperm sorghum reached the United States in 1903 when the Office of Grass and Forage Plant Investigations, U. S. Department of Agriculture, received 255 lots of sorghum seed from India.” This might be the oldest recognition of sugary endosperm in the United States. After, two sugary varieties originating from India were described in detail. The first was P.I.14746 (Kachakachi), which was recognized as an abnormal endosperm type in 1933 in the United States. The second was T.S. I7890 (Vani), a variety of the Surat District of India (Patel and Patel, 1928). The seeds were obtained from B. S. Kadam, a Crop Botanist (Government of Bombay, Karjat, District of Kolaba, India) in 1932, and later, was recognized as a sugary endosperm (Karper and Quinby, 1963). In India, the antiquity of the sugary endosperm character is not known, but the people of India have undoubtedly known of its peculiar sweetness for a long time (Karper and Quinby, 1963). During sorghum breeding in the United States, a mutation from normal to sugary endosperm was recognized at the Chillicothe Station in 1934 in a row of a breeding stock (Blackhull Kafir x Sumac; (Quinby and Karper, 1954)). The three sugary sorghums mentioned above are caused by the same gene based on the results of complementarity tests. This gene pair was later designated as *Su/su* (Karper and Quinby, 1963).

Although a detailed pedigree of SUF was not described, it is possible to estimate that at least one allele (or a maximum of three alleles) existed, based on the literature. Here, however, we found only one allele in NUP in this study. We conducted a molecular genetic analysis of the mutation of SUF, a typical model for sugary sorghum (**Figure 1**). We found that the segregation ratio of F_2_ seeds derived from a cross between MS3B (normal endosperm) and SUF showed a 3:1 ratios, indicating the sugary phenotype was caused by a single recessive gene (**Supplementary Table 1**). These results are consistent with a previous report, that is, the segregation ratio of F_2_ seeds derived from a cross between Kafir (normal endosperm) and Vani (Karper and Quinby, 1963). A subsequent positional mapping identified Sobic.007G204600 (*SbSu*), encoding isoamylase 1 (ISA1), as the corresponding gene for sugary endosperm in SUF (**Figure 2**). Considered the history of sugary sorghum, it is not inconceivable that *SbSu* is *Su* (Karper and Quinby, 1963).

In the starch biosynthesis pathway, ISA1 hydrolyzes α-1,6 glycosidic bonds and removes excess or misplaced branch points in amylopectin introduced by SBEs (Streb et al., 2008). The *isa1* (*su1*) mutants of maize and rice commonly share an endosperm phenotype characterized by wrinkled grains. This phenotype is caused by the accumulation of phytoglycogen, which cannot be stained with iodine solution, and water-soluble sugars in the inner part of the endosperm instead of starch (Pan and Nelson, 1984; James et al., 1995). The previous observations of the *isa1* mutation were also observed in the endosperm of SUF (**Figure 1**), suggesting that a loss of function of *SbSu* causes a similar defect of the starch biosynthesis pathway, especially, debranching in amylopectin.

Gene expression analysis of SUF showed that a loss-of-function of *SbSu* downregulated the expression of essential starch biosynthetic genes encoding the SBEs (*SbSbe1* and *SbSbeIIa*) and SSs (*SbSsI*). Genes encoding DBEs (*SbSu, SbIsa2, SbIsa3*, and *SbPul1*) were initially up-regulated during the early stages of seed development (**Figure 4**). This up-regulation may be due to a mechanism triggered by the decreased ISA1 (SbSU) activity, which may partake in a fine-tuning system for balancing the expression levels of DBEs; this has not been reported before. In the rice *isa1* mutant, the lower expression of most starch synthesis-related genes was observed (Shufen et al., 2019). Similarly, Zhang et al. (2019) reported that the s*e1* mutant, a recessive modifier of *su1* in maize, exhibits decreased expression of many known starch biosynthetic genes (**Figure 4**). Thus, our results indicated that the mutation of *SbSu* induces negative feedback to other DBEs including itself, and subsequently the activity is disappeared in starch synthesis. This may lead to the reduced activity of the pathway, resulting in the accumulation of soluble sugars and wrinkled grains as an end phenotype in SUF (**Figure 1**).

In India, sugary sorghum landraces have been cultivated, however, sugary sorghum varieties from those have not been cultivated outside of India. Two reasons are considered. Firstly, the wrinkled phenotype causes a significant reduction in grain weight (**Figure 1**). Secondly, sugary grains easily become moldy because of the high sugar content and the wrinkled seeds, when it is grown in humid regions. This will lead to decreased grain quality and germination rate. A major reason for these troubles is that only one allele (or some alleles) has been used in the breeding history of sugary sorghum. This could be solved by obtaining moderate to weak alleles for the sugary trait (**Supplementary Figure S5**). In this study, although no haplotype was found showing a sugary trait except for the SUF haplotype in the NUP (**Figure 5**), we found a moderate allele (PI678144) in the EMS mutant panel. This mutation (D182N) is located in a highly conserved region, highlighting the importance of the region for the activity of this DBE (**Supplementary Figure S6**). Moreover, this allele may have potential value in future breeding. In this context, allele mining for other genes related to the sugary trait should also be important. For example, sorghum orthologs of *Sh2* (Sobic.003G230500) and *Se1* (Sobic.005G199300) are reasonable targets, which encode the large subunit of AGPase (Hannah and Nelson, 1976; Bhave et al., 1990) and a monocot-specific protein containing a FANTASTIC FOUR domain with unknown function (Zhang et al., 2019), respectively. Both genes have been incorporated into many sweet corn varieties, including super-sweet corn with improved fresh market quality (Tracy et al., 2019).

Recently, a methodology to create beneficial alleles selected from a continuum of variations in the expression level by genome editing of promoter regions using CRISPR/Cas9 was proposed (Rodriguez-Leal et al., 2017; Cui et al., 2020; Kawai et al., 2020). It is valuable for sorghum breeding to select optimal sugary alleles from a continuum of variations. The findings of this study have aided our understanding of the mechanisms of starch biosynthesis in sorghum and will accelerate breeding using sugary alleles in grain sorghum.

## Data availability statement

The original contributions presented in the study are included in the article/**Supplementary Material**.

## Author contributions

SH, SA-N, and KO-S performed genotyping and plasmid constructions; SH performed rice transformation, histological analysis, and haplotype analysis; SH, HK, and CO performed gene expression analyses; SH, SO, KM, SK, and TS performed field experiments; SH and TS wrote the paper. All authors contributed to the article and approved the submitted version.
