# Supplementary material for: An analysis of sugary endosperm in sorghum: Characterization of mutant phenotypes depending on alleles of the corresponding starch debranching enzyme
Source: Front Plant Sci. 2023 Feb 13;14:1114935. doi: 10.3389/fpls.2023.1114935 (PMC9969085; doi:10.3389/fpls.2023.1114935)
Supplement: Supplementary file 1 [file DataSheet_1.pdf]

Supplementary Material

An analysis of sugary endosperm in sorghum: characterization of mutant phenotypes depending on alleles of the corresponding starch debranching enzyme

Shumpei Hashimoto[1], Satoshi Okada[1], Satoko Araki-Nakamura[1], Kozue Ohmae-Shinohara[1], Kotaro Miura[2], Hideo Kawaguchi[3], Chiaki Ogino[4], Shigemitsu Kasuga[5] & Takashi Sazuka[1]*

*** Correspondence:** Takashi Sazuka; sazuka@agr.nagoya-u.ac.jp

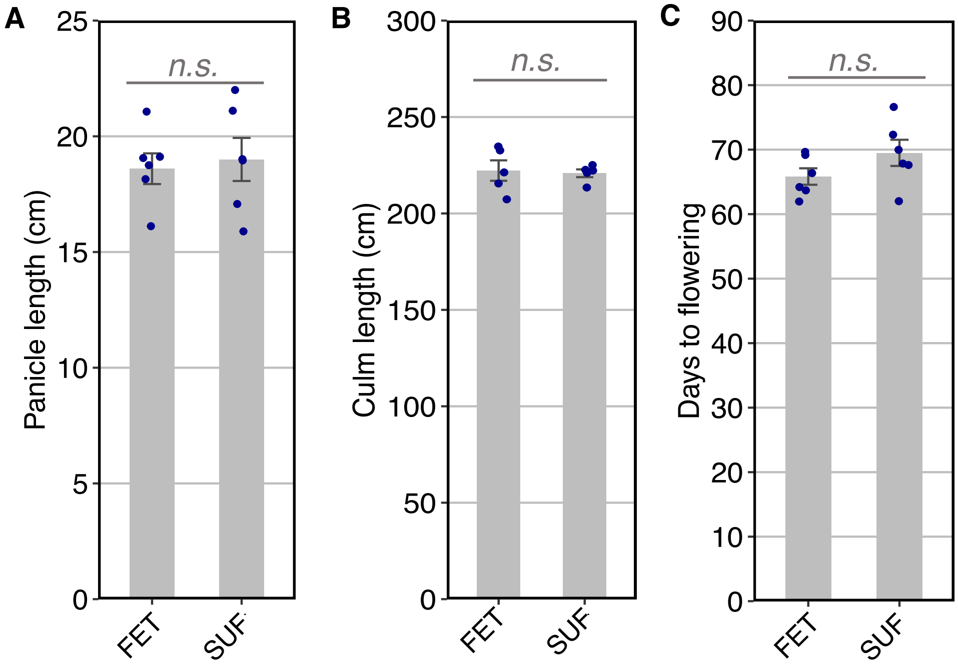

Supplementary Figure S1. Panicle length, culm length, and days to flowering of 'FETERITA'(FET) and 'SUGARY FETERITA'(SUF). FET and SUF were grown in the greenhouse and panicle length (**A**), culm length (**B**), and days to flowering (**C**) were measured (n = 5). All of the trait differences were not statistically significant (n.s., two-tailed t-test).

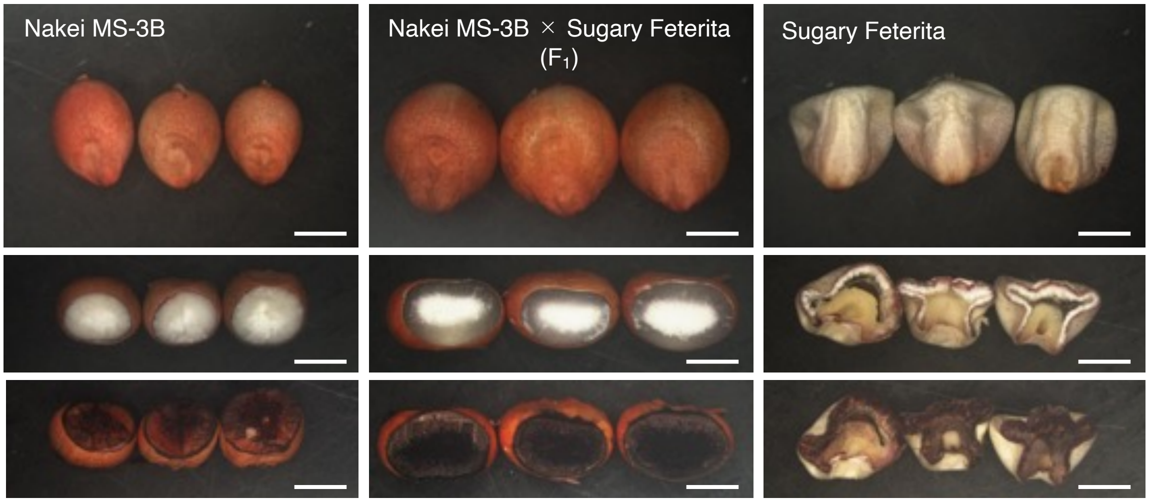

Supplementary Figure S2. The wrinkled phenotype of 'SUGARY FETERITA' (SUF) is recessive. For 'Nakei MS-3B'(MS3B), MS3B × SUF (F_1), and SUF, grain morphologies (upper panels) and crossed sections (middle panels) were observed; the sections were stained with iodine solution (bottom panels). Scale bars: 2 mm.

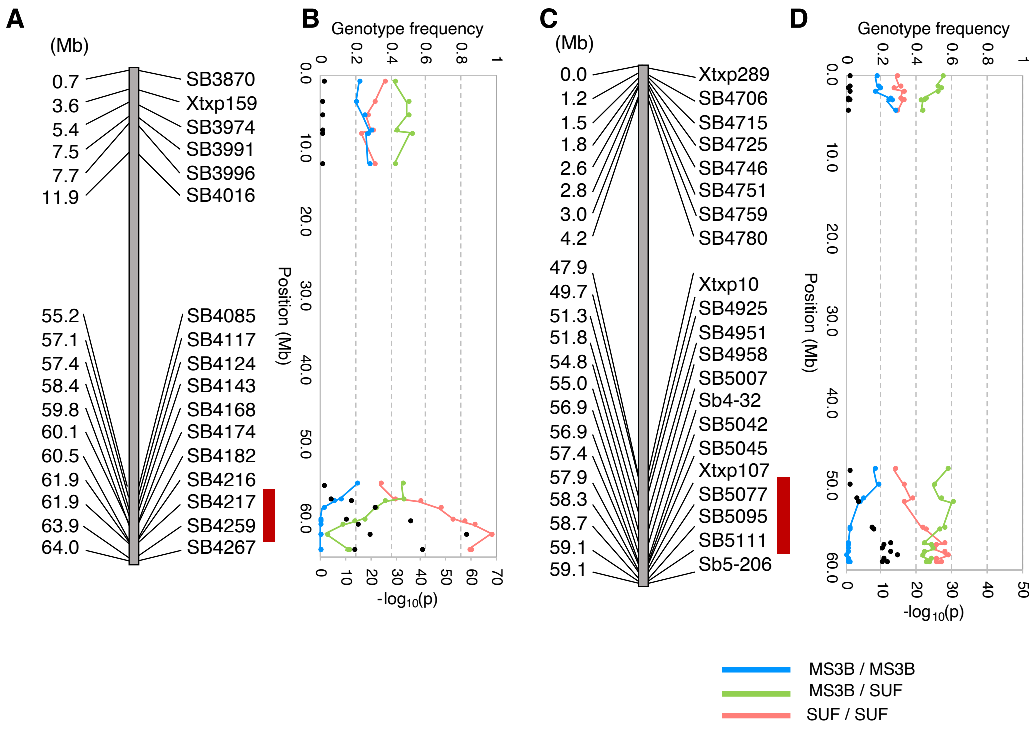

Supplementary Figure S3. Genotype frequencies and segregation ratio per marker in wrinkled F_2 seeds ('Nakei MS-3B' [MS3B] × 'SUGARY FETERITA' [SUF]). For chromosome 7 (**A**) and chromosome 9 (**C**), genomic regions with biased genotype frequencies were detected (red lines) (**B**, **D**). Homozygous MS3B (blue), heterozygous (green), and homozygous SUF (red). Black points indicate $-\log_{10}(p)$ values from the *chi*-square test.

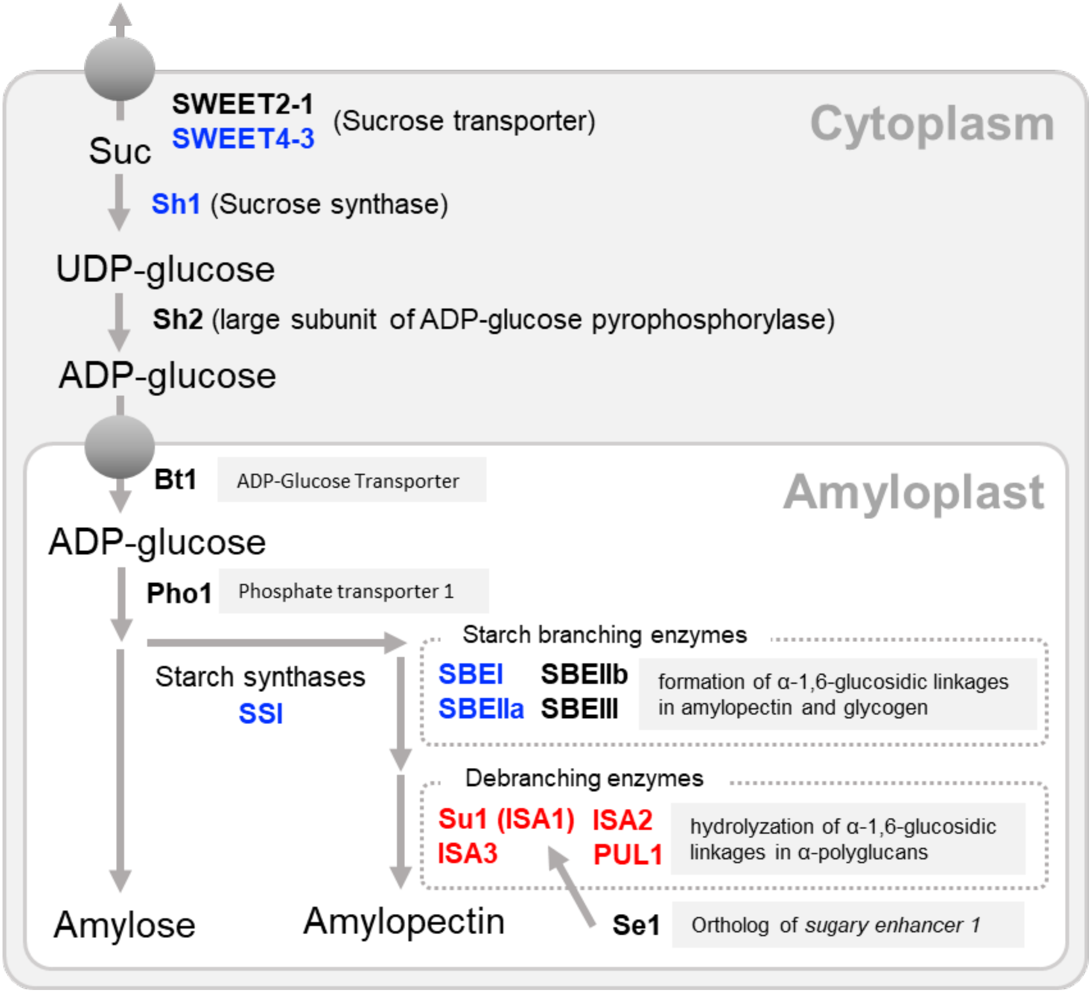

Supplementary Figure S4. The starch synthesis pathway. Genes analyzed by qRT-PCR in this study are shown in this figure. Compared to 'FETERITA', down-regulated and up-regulated genes in 'SUGARY FETERITA' are indicated in blue and red, respectively.

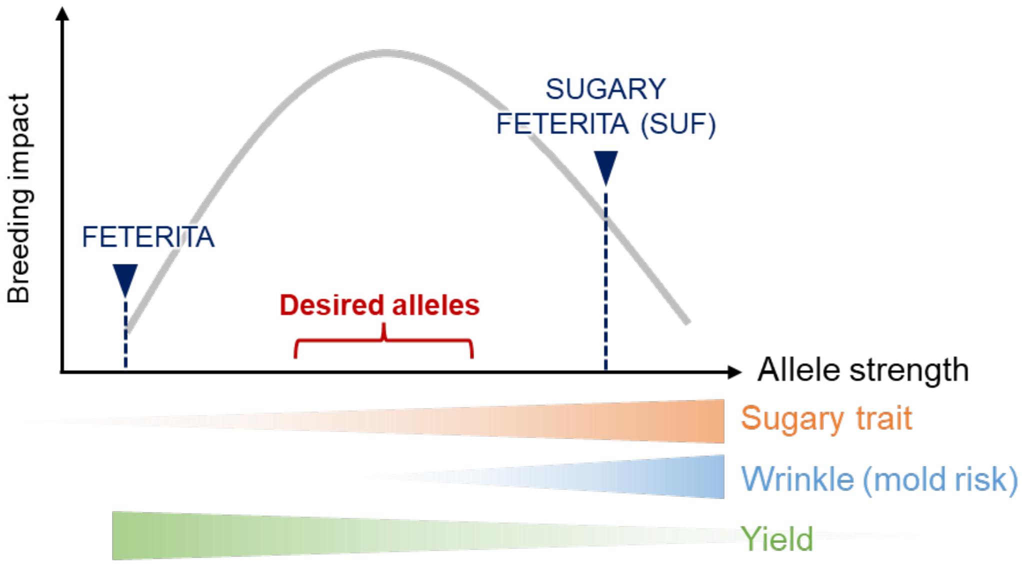

Supplementary Figure S5. Desired alleles for sugary sorghum. The *Su* allele of SUF induces reduced yield and increased risk for mold development. These problems could be solved by moderate alleles for the sugary trait. Here, the allele that exhibits a phenotype that balances reduction in grain weight and mold development with the accumulation of soluble sugars is considered to be the optimal desired allele.

```
                                                  S9A (Hap4)                                          G33V (Hap4,7)  A35C (Hap4)
Sorghum_bicolor_Sobic.007G204600              1 -----MTQLPSVSSPRPLLAVP---------------AVRWRAGVRARPNAAGSAGA-GR   39
Zea_mays_GRMZM2G138060                        1 ----MAQQLPCVSSPRPLLAVP---------------AGRWRAG----VRGRPNVAGLGR   37
Oryza_sativa_LOC_Os08g40930.1                 1 -----MASLPHCLSARPLVVAAAPGRPG-------PGPGPWLRGGARRRNAAFSAGNAGR   48
Arabidopsis_thaliana_AT2G39930.1              1 -----MDAIKCSSSFLHH--------------------TKLNTLFSNHTFPKISAPNF--   33
Aquilegia_coerulea_Aqcoe7G286000.1.p          1 ---MELTQIATHSLLYP----------------------NWRNLKLCPKKDCWNHKQRSG   35
Amborella_trichopoda_XP_006850946             1 --MVSSRSLSCLGSSLSCDICP----------------KKWRS-YSTVTYFRPCGWRFEQ   41
Ginkgo_biloba_Gb_26115                        1 MGFLQCALFTMPGSPYGHAAGP-------------KNFSFCKAGSSKIVAGQQHYGRNEG   47
Selaginella_moellendorffii_EFJ37697.1         1 --------METSTSSYS-------------------------------------------    9
Marchantia_polymorpha_Mapoly0016s0146.1       1 MGVPAAPPFPTASSACLCSAPAPPRVSQYNLSFAFSNSSSAASVFSPLSQFSFNHGADGS   60
Chlamydomonas_reinhardtii_AAP88032.1          1 --MLLQAPGLAPGSARRQAACS-----------VAREATNVRIVTAPALTP-------GR   40

                                                   Q60R (Hap7)    E61D  (Hap7)               E68V (Hap2)
Sorghum_bicolor_Sobic.007G204600             40 GRLFFRAAAARPVATAVQAEQEDGE---------------EEEEVVVEE-------RYAL   77
Zea_mays_GRMZM2G138060                       38 GRLSLHAAAARPVAEAVQAEEDDDD---------------DDEEVAEER--------FAL   74
Oryza_sativa_LOC_Os08g40930.1                49 -RVGLRRSVASAVEVGVGEDEEEGV---------------EEEEEEVEA--VVMPERYAL   90
Arabidopsis_thaliana_AT2G39930.1             34 ----------KPLFRPISISAKDRR------------SNEAENIAVVEK--------PLK   63
Aquilegia_coerulea_Aqcoe7G286000.1.p         36 NGNSFNLKKMKSFVIREKDGGRGGG------------NGSSSSSSSNTEVIETVVIEKPK   83
Amborella_trichopoda_XP_006850946            42 KQVSFPRKRHNLAIMNENGAGDDSE-----------RVKGEPSTVVIER-----------   79
Ginkgo_biloba_Gb_26115                       48 NAKKGHMVTKMEGVRGAVETSKGVG-------------------VFVQE-----------   77
Selaginella_moellendorffii_EFJ37697.1        10 ----------------------------------------SSESVDVKK-----------   18
Marchantia_polymorpha_Mapoly0016s0146.1      61 DGLAGRVNCGGVLYRSPLASGDYIGSGRRKMLTVCGVTGVTSDNASVEKDKVDDGTNQAD  120
Chlamydomonas_reinhardtii_AAP88032.1         41 AGVSGRRILPPSRVVSVELEAPTLS---------------SSPATVSTKKLFCEPSGQPA   85

Sorghum_bicolor_Sobic.007G204600             78 GGAC-RVLAGMPAPLGAT--ALHGGVNFAVYSSGASAASLCLFTPDDLKAETVTEEVPLD  134
Zea_mays_GRMZM2G138060                       75 GGAC-RVLAGMPAPLGAT--ALRGGVNFAVYSSGASAASLCLFAPGDLKADRVTEEVPLD  131
Oryza_sativa_LOC_Os08g40930.1                91 GGAC-RVLAGMPAPLGAT--ALDGGVNFAVYSAGASAASLCLFTPDDLEADEVTEEVPLD  147
Arabidopsis_thaliana_AT2G39930.1             64 SDRF-FISDGLPSPFGPT--VRDDGVNFSVYSTNSVSATICLISLSDLRQNKVTEEIQLD  120
Aquilegia_coerulea_Aqcoe7G286000.1.p         84 LPIF-QVFEGNPMPFGAT--AKDGGVNFAVHSSGAVSATLCLISLSDLQENRVTKKIHLD  140
Amborella_trichopoda_XP_006850946            80 PPRF-NILKGKPWPFGAT--YRDGGVNFALCSNGASSVTLCLMSLSDLQENKTSEQIYLD  136
Ginkgo_biloba_Gb_26115                       78 DLSF-KVLKGSPLPFGAT--ARDGGVNFAVHSSEATSVTLCLFTLSDLQKGIVTKEIPLD  134
Selaginella_moellendorffii_EFJ37697.1        19 --LAGSPKRGRPLPFGAT--PVEGGVNFSVHSSGAIAVSLCLFTEEDLQKGRVSKEFPLH   74
Marchantia_polymorpha_Mapoly0016s0146.1     121 DHICGKPLRGRPLPFGPS--AMNDGVNFSVHSSGASAVSLCLYTEADLEKGQITKEIELH  178
Chlamydomonas_reinhardtii_AAP88032.1         86 STAYGPALTGRPAPLGASIDADTGAINFSVFSSSAESVSLVLFTEADLNAGRATFEIPLD  145

                                                  L139R (Hap2,4,5,6,7,8)                             D182N (PI678144)
Sorghum_bicolor_Sobic.007G204600            135 PLLNLTGNVWHVFIHGDQLHDMLYGYRFDGVFAPER----GQYYDVSNIVVDPYAKAVVS  190
Zea_mays_GRMZM2G138060                      132 PLLNRTGNVWHVFIHGDQLHGMLYGYRFDGVFAPER----GQYYDVSNVVVDPYAKAVVS  187
Oryza_sativa_LOC_Os08g40930.1               148 PLFNRTGNVWHVFIEGE-LHNMLYGYRFDGMFAPHC----GQYFDVSNVVVDPYAKAVIS  202
Arabidopsis_thaliana_AT2G39930.1            121 PSRNRTGHVWHVFLRGD-FKDMLYGYRFDGKFSPEE----GHYYDSSNILLDPYAKAIIS  175
Aquilegia_coerulea_Aqcoe7G286000.1.p        141 PLLNKTGDVWHAFLKGD-FENMLYGYMFDGKFSPEE----GHCYDSSRILLDPYAKAVVS  195
Amborella_trichopoda_XP_006850946           137 PLINKTGDVWHIFLMGN-FEDMLYGYRVDGEFFPEK----GHFFDSLKILLDPYAKAVVS  191
Ginkgo_biloba_Gb_26115                      135 PLVNKTGDVWHAFLRGT-FEDLLYGYQVDGQYDPEE----GHRYDASCILLDPYAKAVIS  189
Selaginella_moellendorffii_EFJ37697.1        75 PVFNRTGDVWHVYLPDV-CPNLLYGYRLNGKFSLEE----GCCYDLSRILVDPYAKAVVS  129
Marchantia_polymorpha_Mapoly0016s0146.1     179 PEFNKTGDVWHVFLPAI-APNLLYGYRVNGRFSPEE----GCCYDSNCILVDPYAKAVIS  233
Chlamydomonas_reinhardtii_AAP88032.1        146 PYVNRTGDVWHIMLPDL-RDDLLYGYRVEGVHQEEDKDYPGMRHDKRRVVLDPYAVAVLN  204

                                                     P199S (Hap7)                                V234I (Hap2,6)
Sorghum_bicolor_Sobic.007G204600            191 RGKYGVPGP----GDN-------CWPQMAGMIPLP-HSTFNWQGDLPLKYHQKDLVIYEM  238
Zea_mays_GRMZM2G138060                      188 RGEYGVPAP----GGS-------CWPQMAGMIPLP-YNKFDWQGDLPLGYHQKDLVIYEM  235
Oryza_sativa_LOC_Os08g40930.1               203 RGEYGVPGP----GGD-------CWPQMAGMIPLP-YSTFDWQGDLPLRYPQKDLVIYEM  250
Arabidopsis_thaliana_AT2G39930.1            176 RDEFGVLGP----DDN-------CWPQMACMVPTR-EEEFDWEGDMHLKLPQKDLVIYEM  223
Aquilegia_coerulea_Aqcoe7G286000.1.p        196 RGEFGALGP----EEN-------CWPQMAGMVPAS-SDEFDWEGDLPLMNPQRDLVIYEM  243
Amborella_trichopoda_XP_006850946           192 RGEYGVPGP----GGD-------CWPQMAGMVPSP-KNDFDWEGDLPLRLPQKDLIIYEM  239
Ginkgo_biloba_Gb_26115                      190 REEYGLLGP----GGN-------CWPQMAGMVPVP-DHKFDWDGDLPLKLPQRDLFIYEM  237
Selaginella_moellendorffii_EFJ37697.1       130 RGKYGALGP----GNS-------CWPQMAGMVPQL-NDEFDWQGDSPPRHKQKDLIIYEL  177
Marchantia_polymorpha_Mapoly0016s0146.1     234 RGLYGMLGP----GGN-------CWPQMAGMVPPLEEDEFDWEGDSPPRLKQKDLIIYEM  282
Chlamydomonas_reinhardtii_AAP88032.1        205 RRRWGQMGPNLPYGEEGVLGVMPTWPQAAAALPAARGSAFDWEGDTPLNLPMESLVIYEA  264

                                            A251T (Hap2,6,7)
Sorghum_bicolor_Sobic.007G204600            239 HLRGFTKHDSSNAKHPGTYIGAVSKLDHLKELGVNCIELMPCHEFNELEYFSSSS-----  293
Zea_mays_GRMZM2G138060                      236 HLRGFTKHNSSKTKHPGTYIGAVSKLDHLKELGVNCIELMPCHEFNELEYFSSSS-----  290
Oryza_sativa_LOC_Os08g40930.1               251 HLRGFTKHSSSNVEHPGTYIGAISKLDYLKELGVNCVELMPCHEFNELEYFSCSS-----  305
Arabidopsis_thaliana_AT2G39930.1            224 HVRGFTRHESSKIEFPGTYQGVAEKLDHLKELGINCIELMPCHEFNELEYYSYNTILGDH  283
Aquilegia_coerulea_Aqcoe7G286000.1.p        244 HVRGFTRHDSSRSNFPGTYLGVVEKLDHLKELGVNCIELMPFQEYNELEYFSYNSVLGNY  303
Amborella_trichopoda_XP_006850946           240 HVRGFTKHDSSEANFPGTYRGMVEKLDHLEELGINAIELMPCHEFNELEYYSYNPVLDDH  299
Ginkgo_biloba_Gb_26115                      238 HVRGFTKHNSSKVDYPGTYHGLVEKLEHLKELGVNAIELMPSQEFNELEYYSYNPVMGDY  297
Selaginella_moellendorffii_EFJ37697.1       178 HMRGFTQHPSSNVDFPGTYLGALEKLPHLKDLGINAIELMPIHEFNELEYYSYNPVMGDH  237
Marchantia_polymorpha_Mapoly0016s0146.1     283 HVRGFTRDSSSGVEHPGTYLGLVEKLPHLKELGVNCIELMPTHEFNELEYYAYNPVVQDH  342
Chlamydomonas_reinhardtii_AAP88032.1        265 HVRGFTAHASSGVAAPGTYAGMVERLDYLKSLGVNAIELLPVFEFNELEYYSQIPGSDQY  324

                                             P308L (PI678119)
Sorghum_bicolor_Sobic.007G204600            294 KMNFWGYSTINFFSPMARYSSS-GIRDSGRGAINEFKTFVREAHKRGIEVIMDVVFNHTA  352
Zea_mays_GRMZM2G138060                      291 KMNFWGYSTINFFSPMARYSSS-GIRDSGCGAINEFKAFVREAHKRGIEVIMDVVFNHTA  349
Oryza_sativa_LOC_Os08g40930.1               306 KMNFWGYSTINFFSPMIRYSSG-GIRNCGRDAINEFKTFVREAHKRGIEVIMDVVFNHTA  364
Arabidopsis_thaliana_AT2G39930.1            284 RVNFWGYSTIGFFSPMIRYASA-SSNNFAGRAINEFKILVKEAHKRGIEVIMDVVLNHTA  342
Aquilegia_coerulea_Aqcoe7G286000.1.p        304 KLNFWGYSTINYFSPMLRYSSA-GIRNCGQDAINEVKILVREAHKRGIEVLMDVVFNHTA  362
Amborella_trichopoda_XP_006850946           300 KVNFWGYSTVNFFSPMMRYSSS-GMQNCGRDAINEFKLLVREAHKRGIEVIMDVVFNHTA  358
Ginkgo_biloba_Gb_26115                      298 KMNFWGYSTVNYFSPMIRYAAS-GNKNCGRDAIKEFKILVREAHKHGIEVFMDVVFNHTA  356
Selaginella_moellendorffii_EFJ37697.1       238 KMNYWGYSTINFFSPMTRYASS-GIQNCGRDAIKEFKTFVREAHKLGIEVFMDVVFNHTA  296
Marchantia_polymorpha_Mapoly0016s0146.1     343 KMNFWGYSTINFFSPMTRYAAS-GSNNCGRDAIKEFKTLIREAHKLGMEVFMDVVFNHTA  401
Chlamydomonas_reinhardtii_AAP88032.1        325 RFNFWGYSTVNYFSPMGRFSAAVGQGAPARASCDEFKQLVKECHRRGIEVILDVVFNHTA  384
```

```
Sorghum_bicolor_Sobic.007G204600              353 EGNEKGPILSFRGIDNSTYYMLAPKGEFYNYSGCGNTFNCNHPVVREFIVDCLRYWVTEM 412
Zea_mays_GRMZM2G138060                        350 EGNEKGPILSFRGIDNSTYYMLAPKGEFYNYSGCGNTFNCNHPVVREFIVDCLRYWVTEM 409
Oryza_sativa_LOC_Os08g40930.1                 365 EGNEKGPILSFRGIDNSTYYMLAPKGEFYNYSGCGNTFNCNHPVVREFIVDCLRYWVTEM 424
Arabidopsis_thaliana_AT2G39930.1              343 EGNEKGPIFSFRGVDNSVYYMLAPKGEFYNYSGCGNTFNCNHPVVRQFILDCLRYWVTEM 402
Aquilegia_coerulea_Aqcoe7G286000.1.p          363 EGNEKGPTLSFRGVDNSVYYMLAPKGEYYNYSGCGNTFNCNHPVVRQFIVDCLRYWVEEM 422
Amborella_trichopoda_XP_006850946             359 EGNEKGPTFSFRGLDSCVYYMLAPKGEFYNYSGCGNTVNCNHPVVRQFIVDCLRYWVTEM 418
Ginkgo_biloba_Gb_26115                        357 EGNEMGPILSFRGLDNSVYYMLAPKGEFYNYSGCGNTFNCNHPVVRRFIVDCLRYWVIEM 416
Selaginella_moellendorffii_EFJ37697.1         297 EGNEMGPIISFRGLDNRVYYMTAPKGEFYNYSGCGNTFNCNHPVVRRFIVDCLRYWVLEM 356
Marchantia_polymorpha_Mapoly0016s0146.1       402 EGNEMGPTISFRGFDNRVYYMIAPKGEFYNYSGCGNTFNCNHPIVRRFIIDCLRYWVLEM 461
Chlamydomonas_reinhardtii_AAP88032.1          385 EGNERGPTISFRGLDNRVYYMLAPGGEYYNYSGCGNTLNCNQPVVRQFILDCLKHWVTEY 444

                                                           ▼L421H (SUF)
Sorghum_bicolor_Sobic.007G204600              413 HVDGFRFDLASIMTRGCSLWDPVN-----------------VYGSP--MEGDMITTGTPL 453
Zea_mays_GRMZM2G138060                        410 HVDGFRFDLASILTRGCSLWDPVN-----------------VYGSP--MEGDMITTGTPL 450
Oryza_sativa_LOC_Os08g40930.1                 425 HVDGFRFDLASIMTRGCSLWDPVN-----------------VYGSP--VEGDMTTTGTPL 465
Arabidopsis_thaliana_AT2G39930.1              403 HVDGFRFDLGSIMSRSSSLWDAAN-----------------VYGAD--VEGDLLTTGTPI 443
Aquilegia_coerulea_Aqcoe7G286000.1.p          423 HVDGFRFDLAAILTRSSSLWDAVN-----------------VYGNS--IEGDSLTTGTPL 463
Amborella_trichopoda_XP_006850946             419 HVDGFRFDLASIMTRSCSLWDAAN-----------------AFGSP--IEGHSITTGTPL 459
Ginkgo_biloba_Gb_26115                        417 HVDGFRFDLASIMTRACSLWDRTN-----------------VFGNV--IEGDFLTTGTPL 457
Selaginella_moellendorffii_EFJ37697.1         357 HIDGFRFDLASILTRASSLWDKAN-----------------VFGTSEDVDGDSVTTGTPL 399
Marchantia_polymorpha_Mapoly0016s0146.1       462 HVDGFRFDLASILTRASSLWDREN-----------------VFGSPEGPEGDVVTTGTPL 504
Chlamydomonas_reinhardtii_AAP88032.1          445 HVDGFRFDLASILTRAHSAWHPQQYDQETGQRVAMSSGGAIVTAEGIMTDGAGVPTGYPL 504

Sorghum_bicolor_Sobic.007G204600              454 VSPPLVDMISNDPILGDVKLIAEAWDAGGLYQVGQFPHW-NVWSEWNGKYRDTVRQFIKG 512
Zea_mays_GRMZM2G138060                        451 VAPPLIDMISNDPILGNVKLIAEAWDAGGLYQVGQFPHW-NVWSEWNGKYRDTVRQFIKG 509
Oryza_sativa_LOC_Os08g40930.1                 466 ATPPLIDMISNDPILGDVKLIAEAWDAGGLYQVGQFPHW-KIWSEWNGKYRDIVRQFIKG 524
Arabidopsis_thaliana_AT2G39930.1              444 SCPPVIDMISNDPILRGVKLIAEAWDAGGLYQVGMFPHW-GIWSEWNGKFRDVVRQFIKG 502
Aquilegia_coerulea_Aqcoe7G286000.1.p          464 GSPPLIDMISNDPILRRVKLIAEAWDTGGLYQVGMFPHW-GIWSEWNGKYRDIVRQFIKG 522
Amborella_trichopoda_XP_006850946             460 SNPPLIDLISNDPVLNGIKLIAEAWDAGGLYQVGGFPHH-GVWSEWNGQYRDIVRQFIKG 518
Ginkgo_biloba_Gb_26115                        458 NEPPLIDMISNDPVLHGIKLIAEAWDIGGLYQVGSFPHW-GIWSEWNGQYRDTVRQFIKG 516
Selaginella_moellendorffii_EFJ37697.1         400 SEPPLIDMISNDPVLRGVKLIAEAWDSGGLYQVGNFPHW-GIWSEWNGQYRDTVRLFIKG 458
Marchantia_polymorpha_Mapoly0016s0146.1       505 SEPPLVDMMSNDPVLRGVKLIAEAWDTGGLYQVGSFPHW-GVWSEWNGQFRDTVRSFIKG 563
Chlamydomonas_reinhardtii_AAP88032.1          505 ADPPLVESISEDPVLRNTKMIAEAWDCDGLNQVGAFPHYGGRWSEWNGKFRDVVRNFIKG 564

Sorghum_bicolor_Sobic.007G204600              513 TDG-FAGAFAECLCGSPQLYQA------------------GGRKPWHSVNFVCAHDGFTL 553
Zea_mays_GRMZM2G138060                        510 TDG-FAGAFAECLCGSPQLYQA------------------GGRKPWHSINFVCAHDGFTL 550
Oryza_sativa_LOC_Os08g40930.1                 525 TDG-FAGGFAECLCGSPHLYQA------------------GGRKPWHSINFVCAHDGFTL 565
Arabidopsis_thaliana_AT2G39930.1              503 TDG-FSGAFAECLCGSPNLYQ-------------------GGRKPWHSINFICAHDGFTL 542
Aquilegia_coerulea_Aqcoe7G286000.1.p          523 TDG-FSGAFAECLCGSPNLYQ-------------------GGRKPWNSINFVCAHDGFTL 562
Amborella_trichopoda_XP_006850946             519 TDG-LSGVFAECLCGSPQLYQG------------------GGRKPWNSINFICAHDGLTL 559
Ginkgo_biloba_Gb_26115                        517 TDG-LAGAFAQCLCGSPHLYQE------------------GGRKPWHSINFVSAHDGFSL 557
Selaginella_moellendorffii_EFJ37697.1         459 TDG-LAGAFAQCLCGSPHLYQD------------------GGRKPWHSVNFVTAHDGFTL 499
Marchantia_polymorpha_Mapoly0016s0146.1       564 TDG-MVGLFAQCLCGSPHLYQE------------------SGRKPWHSVNFVTAHDGFTL 604
Chlamydomonas_reinhardtii_AAP88032.1          565 TDGPWAGDFASAICGSPNIYANNTPHETDWWANNGGRQWKGGRGPHASINFVAAHDGFTL 624

Sorghum_bicolor_Sobic.007G204600              554 ADLVTYNNKYNLSNGENNRDGENHNLSWNCGEEGEFASLSVRRLRKRQMRNFFVCLMVSQ 613
Zea_mays_GRMZM2G138060                        551 ADLVTYNSKYNLSNGEDNRDGENHNLSWNCGEEGEFASLSVRRLRKRQMRNFFVCLMVSQ 610
Oryza_sativa_LOC_Os08g40930.1                 566 ADLVTYNKKYNSSNGEDNRDGENHNLSWNCGEEGEFAGLSVKRLRKRQMRNFFVSLMVSQ 625
Arabidopsis_thaliana_AT2G39930.1              543 ADLVTYNNKNNLANGEENNDGENHNYSWNCGEEGDFASISVKRLRKRQMRNFFVSLMVSQ 602
Aquilegia_coerulea_Aqcoe7G286000.1.p          563 ADLVTYNNKHNLANGEDNNDGETHNNSWNCGQEGEFASLSVKRLRKRQMRNFFLCLMVSQ 622
Amborella_trichopoda_XP_006850946             560 SDLVSYNEKHNSANGEGNNDGEDHNNSWNCGQEGEFASLPVQRLRKRQMRNFFLCLMVSQ 619
Ginkgo_biloba_Gb_26115                        558 ADLVSYNQKHNSANGEDNNDGEDHNNSWNCGEEGEFVSLPVQHLRQRQMRNFIVCLMVSQ 617
Selaginella_moellendorffii_EFJ37697.1         500 LDLVSYNSKHNIANGESNNDGETHNNSWNCGEEGELVSIRVRRLRHRQLRNFLVALMVSQ 559
Marchantia_polymorpha_Mapoly0016s0146.1       605 ADLVSYNEKHNEANGENNNDGESHNASWNCGEEGDTHNFSVLRLRQRQLRNFMLALMVSQ 664
Chlamydomonas_reinhardtii_AAP88032.1          625 ADMVAYNNKHNEANGENNRDGEQHNNSWNCGEEGPTTKWEVNRLRQRQMRNLTGALLLSC 684

Sorghum_bicolor_Sobic.007G204600              614 GVPMFYMGDEYGHTKGGNNNTYCHDHYVNYFRWDKKEEQSSDLYRFCRLMTKFRKECESL 673
Zea_mays_GRMZM2G138060                        611 GVPMFYMGDEYGHTKGGNNNTYCHDHYVNYFRWDKKEEQSSDLYRFCRLMTKFRKECESL 670
Oryza_sativa_LOC_Os08g40930.1                 626 GVPMFYMGDEYGHTKGGNNNTYCHDHYVNYFRWDKKEE-SSDLQRFCSLMTKFRKQCESL 684
Arabidopsis_thaliana_AT2G39930.1              603 GVPMIYMGDEYGHTKGGNNNTYCHDNYMNYFRWDKKEEAHSDFFRFCRILIKFRDECESL 662
Aquilegia_coerulea_Aqcoe7G286000.1.p          623 GVPMIYMGDEYGHTKGGNNNTYCHDNYINYFRWDKKEESSNDFFRFCCLMTKFRQECESL 682
Amborella_trichopoda_XP_006850946             620 GTPMIFMGDEYGHTKGGNNNTYCHDSYINYFRWDKKEDASSDFFRFCRLMTKFRRECESL 679
Ginkgo_biloba_Gb_26115                        618 GIPMLTMGDEYGHTKGGNNNTYCHDNYVNYFRWDKKEASQTGFFRFCTLMSKLRSECESL 677
Selaginella_moellendorffii_EFJ37697.1         560 GVPMVTMGDEYAHTKGGNNNTYCHDNAINYFRWDKLRTDPTGLFRFSRHLFNFRRQ--SL 617
Marchantia_polymorpha_Mapoly0016s0146.1       665 GVPMVTMGDEYGHTKGGNNNSYCHDNIINYFQWDQKNLDAQGFYRFSRLLINFRRQCDAL 724
Chlamydomonas_reinhardtii_AAP88032.1          685 GVPMINMGDEYGHSKNGNNNTYCHDSELNYLRWDQLAEDPHGFNRFVRLLIHFRRATPAL 744

                                                                                ▼R701Q (PI678151)
Sorghum_bicolor_Sobic.007G204600              674 GLEDFPTSERLQWHGHQPGKPDWSEASRFVAFTMKDETKGEIYVAFNTSHLPVVVGLPER 733
Zea_mays_GRMZM2G138060                        671 GLEDFPTSERLKWHGHQPGKPDWSEASRFVAFTMKDETKGEIYVAFNTSHLPVVVGLPER 730
Oryza_sativa_LOC_Os08g40930.1                 685 GLADFPTAQRLHWHGHQPGKPDWSETSRFVAFSTKDETKGEIYVAFNASHLPAVVGLPER 744
Arabidopsis_thaliana_AT2G39930.1              663 GLNDFPTAKRLQWHGLAPEIPNWSETSRFVAFSLVDSVKKEIYVAFNTSHLATLVSLPNR 722
Aquilegia_coerulea_Aqcoe7G286000.1.p          683 GLDDFPTADRLQWHGHSPGVPDWSETSRFVAFSLIDSVKGEIYLAFNTSHLPVTVSLPER 742
Amborella_trichopoda_XP_006850946             680 GLDDFPTGERLQWHGHSPGVPDWSESSRFVAFSLMGSIEGEIYVAFNTSHLPVTIELPER 739
Ginkgo_biloba_Gb_26115                        678 GLEEFPTAERLQWHGHLPEIPDWSESSRFVAFTIVDSIKGELYIAFNTSHLPVTVTLPER 737
Selaginella_moellendorffii_EFJ37697.1         618 GLGDFPTAERLEWHGVVPETPDWSETSRFVAFSLVDAKKRELYIAFNTSHLPVLVTLPER 677
Marchantia_polymorpha_Mapoly0016s0146.1       725 RLGEFPTAEKLQWHGSVPNLPDWSDSSRFVAFSLVDSQSKELYVAFNASHLPTVVSLPER 784
Chlamydomonas_reinhardtii_AAP88032.1          745 QRTTFVNDKDIQWHGELPNTPDWTDTSRLVAFTLHDGKGGGLYVAFNTSHLPKLLQLPKW 804
```

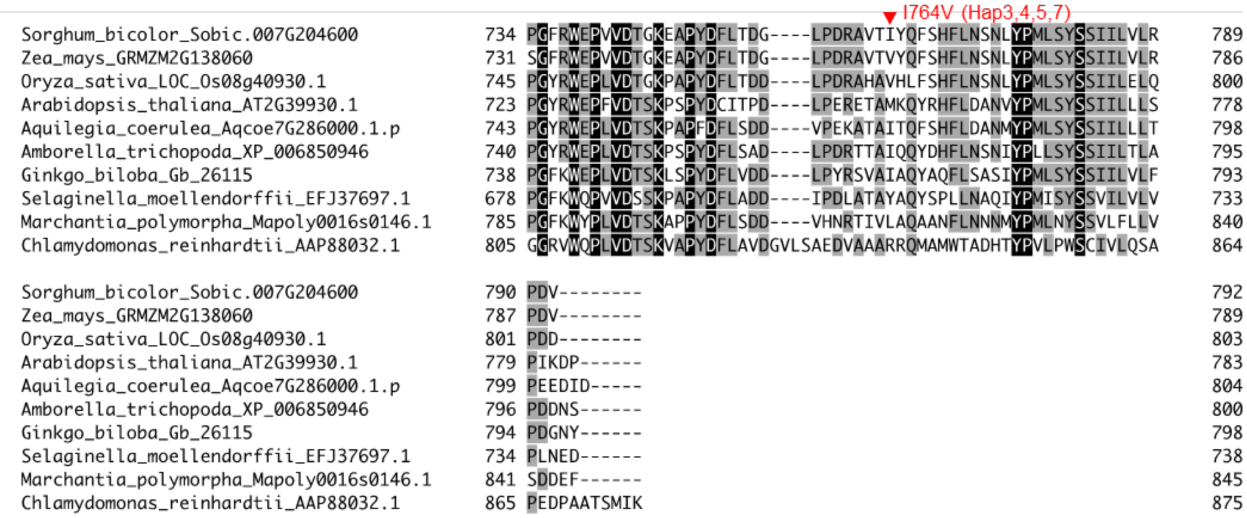

Supplementary Figure S6. Alignment of SbSU homologs. *SbSU* homologs of *Zea mays, Oryza sativa, Arabidopsis thaliana, Aquilegia coerulea, Amborella trichopoda, Ginkgo biloba, Selaginella moellendorffii, Marchantia polymorpha*, and *Chlamydomonas reinhardtii* were obtained from the relevant databases (Phytozome 13: https://phytozome-next.jgi.doe.gov/, NCBI: https://www.ncbi.nlm.nih.gov/, Ginkgo DB: https://ginkgo.zju.edu.cn/genome) by BLASTp and aligned with MUSCLE using the GENETYX-MAC software (v20.1.0). The regions highlighted in blue and yellow indicate the carbohydrate-binding module and catalytic domain, respectively.

Supplementary Table S1. Segregation ratio of the sugary phenotype in the F_2 progeny resulting from a cross between 'Nakei MS-3B' and 'Sugary feterita'.

Cross-combination	Segregation		Total	p value (χ^2 test)
	Normal	Sugary		
Nakei MS-3B × Sugary Feterita	1212	415	1627	0.63

Supplementary Table S2. Sorghum accessions examined in this study.

Accession / variety	Origin	Haplotype of *SbSu1*
E 9	Chad	Hap_1
E 17	Chad	Hap_1
SC 112	Ethiopia	Hap_1
THIBA RED	Ethiopia	Hap_1
GIZA 3/59	Ethiopia	Hap_1
MN 4566	Ethiopia	Hap_1
Nerum Boer	Ethiopia	Hap_1
MERASI	Ethiopia	Hap_1
AKLMOI WHITE	Kenya	Hap_1
E 959	Kenya	Hap_1
Nyagwang No.56	Kenya	Hap_1
Malwal Tonj	Kenya	Hap_1
MN2014	Malawi	Hap_1
AIT BRAHIM	Morocco	Hap_1
PHATSAI	Morocco	Hap_1
ZA113 DAWA PAS PARA	Nigeria	Hap_1
E 1089	Sudan	Hap_1
HEGARI MALOWAR	Sudan	Hap_1
E 1091	Sudan	Hap_1
LAMBAS	Sudan	Hap_1
240 WAD UMM BENEIN	Sudan	Hap_1
Duro El Jack	Sudan	Hap_1
No.5 GAMBELA	Sudan	Hap_1
Dura Huria	Sudan	Hap_1
PI 643016	Sudan	Hap_1
Red Losinga	Sudan	Hap_1
WAD FUR WHITE	Sudan	Hap_1
PI152959	Sudan	Hap_1
S.A.1	Swaziland	Hap_1
S.A.2	Swaziland	Hap_1
MN 2363	Tanzania	Hap_1
AS 4637 NHORONGO NENPI	Tanzania	Hap_1
E 37	Tanzania	Hap_1
MN2277	Tanzania	Hap_1
MN 2363	Tanzania	Hap_1
E 276 FRAMIDA	Uganda	Hap_1
S. VULGARE 72-726-7	Uganda	Hap_1
L31 Emiroit	Uganda	Hap_1
Serere	Uganda	Hap_1
GRASSL	Uganda	Hap_1
U.T.23	Zaire	Hap_1
BIG WHITE HULL	China	Hap_1
IS 13726	China	Hap_1
CHINESE AMBER	China	Hap_1
MN3080	China	Hap_1
Y. E. (I. P.) INT. TYPE	India	Hap_1

Accession / variety	Origin	Haplotype of *SbSu1*
DHOOTI ANEHULA	India	Hap_1
DELHI LOCAL	India	Hap_1
SA 9804 BIG SEED Y.E. (JP360)	India	Hap_1
SANGOD	India	Hap_1
M.35-1 DODDA MAGADI	India	Hap_1
R.473	India	Hap_1
HONEY SORGHUM	India	Hap_1
IKEDACHO MATSUO ZAIRAI	Japan	Hap_1
SIL	Japan	Hap_1
MN 4138,IS 21863	Japan	Hap_1
PI 229486 VULGARE	Iran	Hap_1
COL/PAK/1991/IBPGR/2724(2)	Pakistan	Hap_1
COL/PAK/1989/IBPGR/2427(5)	Pakistan	Hap_1
COL/PAK/1989/IBPGR/2420(1)	Pakistan	Hap_1
COL/PAK/1989/IBPGR/2550(1)	Pakistan	Hap_1
IS 2352	Pakistan	Hap_1
IS 12893	Syria	Hap_1
IS 21863	Syria	Hap_1
MN2826	Turkey	Hap_1
CHOONCHAN LOCAL	Korea	Hap_1
MOCTAC LOCAL	Korea	Hap_1
HANGETSUTOSUI	Korea	Hap_1
KOUSHUU ZAIRAISHU	Korea	Hap_1
KOUBOUSHI	Korea	Hap_1
TRACY	Australia	Hap_1
IS 27818	Hungary	Hap_1
MN4135	Jugoslavija	Hap_1
NYAKASOBA BEST	Lesotho	Hap_1
MAKHOTLONG I	Lesotho	Hap_1
TENANT WHITE	Lesotho	Hap_1
AW 70/12 DL/59/1532	South Africa	Hap_1
E 232 INGWARUMA PEARLY	South Africa	Hap_1
RED KAFIR	South Africa	Hap_1
EAR FROM PIETESBURG DL/60/107	South Africa	Hap_1
S.BASUTORUM DL/60/97	South Africa	Hap_1
MN 2680	South Africa	Hap_1
Inyangentombi	South Africa	Hap_1
Manyoble	South Africa	Hap_1
Tugela Ferry	South Africa	Hap_1
TEGEVINI	South Africa	Hap_1
CAPE COLO 28/53	Zimbabwe	Hap_1
BATTANBAN	Cambodia	Hap_1
AS 5781 HUAN SA PHAUNG AH LPYSU	Myanmar	Hap_1
JUNELO	Nepal	Hap_1
NM3095	Taiwan	Hap_1
Chohin 232	Unknown	Hap_1

Accession / variety	Origin	Haplotype of *SbSu1*
MS138B	Unknown	Hap_1
MS 175A	Unknown	Hap_1
M36001	Unknown	Hap_1
FETERITA WAD UMM BENEIN	Unknown	Hap_1
FETERITA FAYOUMI D.S.21	Unknown	Hap_1
PI302236	Unknown	Hap_1
PI302173	Unknown	Hap_1
KELLER	USA	Hap_1
Dale	USA	Hap_1
EARLY FOLGER	USA	Hap_1
PLANTER	USA	Hap_1
DALE	USA	Hap_1
TOP 76-6	USA	Hap_1
SMITH	USA	Hap_1
Theis	USA	Hap_1
EARLY SUMAC	USA	Hap_1
Grif 16016	USA	Hap_1
COLLIER	USA	Hap_1
WRAY	USA	Hap_1
PI658665	USA	Hap_1
Rio	USA	Hap_1
Theis	USA	Hap_1
WRAY	USA	Hap_1
MN 401	Algeria	Hap_2
MN 4566	Ethiopia	Hap_2
PI 329762	Ethiopia	Hap_2
IS 2131	Ethiopia	Hap_2
MN4578	Ethiopia	Hap_2
MN 2089	Malawi	Hap_2
CHEDOMBA	Malawi	Hap_2
MN 2089	Malawi	Hap_2
KOURNIANIA	Morocco	Hap_2
KA 24	Nigeria	Hap_2
MN 1277 MUHEYAR	Nigeria	Hap_2
109 TONJI	Sudan	Hap_2
MALNAL	Sudan	Hap_2
Ayuak	Sudan	Hap_2
Feterita Abu Derega	Sudan	Hap_2
MN1644	Tanzania	Hap_2
UGANDA L 1	Uganda	Hap_2
LIAZOA 1	China	Hap_2
Daikokaku	China	Hap_2
SWEET JOWAR SEEDED 263	India	Hap_2
MAJEVARI	India	Hap_2
GOOSENECK	India	Hap_2
RABI YANGAR JORA MITHUGADUR	India	Hap_2
SWEET JOWAR SELECTED	India	Hap_2

Accession / variety	Origin	Haplotype of *SbSu1*
KAMANDRI	India	Hap_2
PI 179749	India	Hap_2
B.35	India	Hap_2
Juar(PI 180487)	India	Hap_2
Chohin 224	Japan	Hap_2
KASSHOKU ZAIRAI-SHU	Japan	Hap_2
HATASHIKI ZAIRAI	Japan	Hap_2
KANAGAWA ZAIRAI	Japan	Hap_2
OOTOYO-MURA ZAIRAI	Japan	Hap_2
KIKUCHI ZAIRAI	Japan	Hap_2
TAKAKIMI	Japan	Hap_2
PI 220636 Q 2/3/56	Afghanistan	Hap_2
COL/PAK/1989/IBPGR/2592(7)	Pakistan	Hap_2
COL/PAK/1989/IBPGR/2411(1)	Pakistan	Hap_2
87-9-21-3-1	Pakistan	Hap_2
87-9-21-3-2	Pakistan	Hap_2
COL/PAK/1989/IBPGR/2553(4)	Pakistan	Hap_2
Dhurra No.7	Yemen	Hap_2
ESHOME	South Africa	Hap_2
WAD YABOO 132/53	Zimbabwe	Hap_2
EC 18868	Nepal	Hap_2
PI199869	Unknown	Hap_2
PI302199	Unknown	Hap_2
60 DAY MILO(60M)	USA	Hap_2
38 DAY MIRO(38M)	USA	Hap_2
RYER MILO(44M)	USA	Hap_2
SM 100 MILO	USA	Hap_2
100 DAY MILO(100M)	USA	Hap_2
90 DAY MILO(90M)	USA	Hap_2
Grif 16016,MN 2751	USA	Hap_2
KELLER	USA	Hap_2
M 81E	USA	Hap_2
143 DINDERAWI 1	Sudan	Hap_3
DINDERAWI 1	Sudan	Hap_3
NAGAD EL MUR	Sudan	Hap_3
Greenleaf Sudan9	Sudan	Hap_3
HIMEKI ZAIRAI	Japan	Hap_3
MS79B	Unknown	Hap_3
LAHOMA(JUKEI SUDAN GRASS)	Unknown	Hap_3
Brawley	USA	Hap_3
MN4254	USA	Hap_3
SCHROCK	Morocco	Hap_4
RAHMETALLA GALLABAT	South Africa	Hap_4
COLMAN(Y)	USA	Hap_4
HONEY DRIP	USA	Hap_4
Jerima	Nigeria	Hap_5
PI213901	Unknown	Hap_6
PI302136	Unknown	Hap_7

Supplementary Table S3. Primers used in this study.

Name	Forward primer (5' to 3')	Reverse primer (5' to 3')	Purpose
SB72	CTTCCAGCACTACCTCGTCATGC	AGGAAGCTCTGGATCAGCGTGTT	Mapping
SB134	CCCAAATTAAGCTGAGATGATGGC	CCATAAAATTGTTCCACGGTTCTCA	Mapping
SB169	AGTGGCAACTGGCAGTATTTGACA	ATCGGAAGTGCAGACACTCACAAC	Mapping
SB369	GAGGAAGGATGACAAAGAGGGTGA	GGAAAAAGCACATTGCTGACTCCT	Mapping
SB491	GACGAAGAAGAGCTTAGCGTGGAG	GTAGATGAACCTGGTGTGCTTCCC	Mapping
Xtxp32	AGAAATTCACCATGCTGCAG	ACCTCACAGGCCATGTCG	Mapping
Xtxp11	TCGAGAAATTCAACATGCTG	GCTAGACCGACGAGATAAG	Mapping
Xtxp37	AACCTAAGAGGCCTATTTAACC	ACGGCGACTATGTAACTCATAG	Mapping
Xtxp335	TATTTCCTCTTGAAAGAATCAGGG	TATTCATCGAGCAAAAGGCA	Mapping
Xtxp75	CGATGCCTCGAAAAAAAAACG	CCGATCAGAGCGTGGCAGG	Mapping
Xtxp61	GATGCCCATGCCTTGC	CCCACTAAACTAAAGCGGACA	Mapping
Xtxp319	TAGACATCTGAATTAAGGAGC	CATGCCCCTGAAAGAGA	Mapping
Xtxp248	GGGTGTCCAATGTTGTCTGC	GGCCGTTACTGTCCCTTACTCA	Mapping
Xtxp323	TATATGCATGTTTTAGGTCG	CTGTTGTTCCTTTCTTCC	Mapping
Xcup53	GCAGGAGTATAGGCAGAGGC	CGACATGACAAGCTCAAACG	Mapping
SB960	GGCATGACATTCTTTCTAGCCCTG	GAAGGAGTCATTGGCCAAATCGTA	Mapping
Xtxp197	GCGTCAATTAATCCAAACAGCCTC	GAGTTCCTATTCCCGTTCATGGTGAT	Mapping
SB969	TTTGTTCATCTCACACGAAACCGT	TGAAATTAGGCACCCAATTCAACC	Mapping
SB992	CAACAACATCAACACTCCCTCCAA	ACCACCATCATCATGGCGTCC	Mapping
SB1001	CGGGTACTTGGAGATCGAAGAAAA	AAAAACACGAAAGGAAACTCGCTG	Mapping
Xtxp297	GACCCATATGTGGTTTAGTCGCAAAG	GCACAATCTTCGCCTAAATCAACAAT	Mapping
Xtxp21 1	TCAACGGCCAATGATTTCTAAC	AGGTTGCGAATAAAAGGTAATGTG	Mapping
SB1048	TAGCATCTCCAACAGCATCCTCAA	ACGTGGCAATTTGTGGGTCTACTC	Mapping
SB1094	AGCTGTCTTCCTTCCTTCTCCTCC	CGTACAAAGCAAACGATTTTGCAC	Mapping
SB1109	CTCTCACACACACACGCACACTTT	AATCTTGGAGTTCCTCCCTGCTTC	Mapping
Xtxp3	TCTTTCCCAAGGAGCCTAG	GAAGTTATGCCAGACATGCTG	Mapping
Xtxp72	TTATGGAAGCAAAATGAC	CGAATCCTAATTGAGGTAAGC	Mapping
Xtxp55	TCATGGCATGGGACTATTG	AAGGTTGGCGTAGAAATGTGT	Mapping
SB1176	CGTACGTGCTTCTAGCTCTCGACT	GTTTCAATCATTCATTCAACCGCC	Mapping
SB1186	GGCTGATATGATCAAGCTGGTTGT	AGGGATCGAGGCCCTACCTAATTT	Mapping
Xtxp298	GCATGTGTCAGATGATCTGGTGA	GCTGTTAGC7CTTCTAATCGTCGGT	Mapping
SB1264	CTCAGTTCACGACGATTTGACTGC	ACCACGCATCTTTTCGTAGGTCAT	Mapping
SB1292	AGCTGCTACAGAGACAGCACCAAA	CCCCCTTCC7CTCATATAGCACC	Mapping
Xtxp1	TTGGCTTTTGTGGAGCTG	ACCCAGCAGCACTACACTAC	Mapping
SB1369	GCCCTTTCTTACCACAGCCAATATC	TAGTTTTCTTGGGAAGGATGAGCG	Mapping
Xtxp7	ACATCTACTACCCTCTCACC	ACACATCGAGACCAGTTG	Mapping
SB1493	AAGTTTTCGCAAAATGGCTTACCC	GGGGTGTGATATATGCAAAGGGAA	Mapping
SB1549	ACGGACACGATCGAGATGCTAAAT	CCCCTTCATTCCTCGTCTCTCTCT	Mapping
SB1554	GTTTTGGCGTCACAAAGCTTATCC	CGAGATTCAGGCCACGTAAACTCT	Mapping
SB1562	GAGACGACGCTAATCCATCCAAAC	GCAAGAACACCAGTGCATACATCC	Mapping
Xtxp8	ATATGGAAGGAAGAAGCCGG	AACACAACATGCACGCATG	Mapping
SB1658	ACAAGTGGCGTAGCATCATCCATA	AATCTGACCGAGGAGACGACTGAC	Mapping
SB1697	GCGGATGATCTTTCTCAGCTCTTC	GGTAGCCAACAGCATTGCCATATT	Mapping
SB1714	CCTAATCATCTCGCTGTTGCATTG	TCATAGGGCCTGAAAAATCAGGAA	Mapping
SB1749	GGAAAATAGGCTGATGAGGCTGAA	GGGGCAACACAAAGTTACGACTTC	Mapping
SB1764	CTTG+A16TGCTTGCTTGCACCATATTC	GTCGATGAGGAGCTTCATGCTCAG	Mapping
SB1799	GTCACTTGGCATAATCCATCCCAT	CGGGTTATGTCGGTGTATGTAGCA	Mapping
SB1814	TTTAATCCAATCCACGGAGAATGC	CGGAAATGATTGCCAGTTTCAGTA	Mapping
SB1834	GGGCAAATACGAGAAGAAAACCAA	TGACGCATCAGAATCAATGGTACA	Mapping
SB1839	TGAAGAAGCTGTCCGTTTGACAAG	AAAAAGCTGCTCCTTTGAGGCTTT	Mapping
Xtxp31	TGCGAGGCTGCCCTACTAG	TGGACGTACCTATTGGTGC	Mapping
SB2069	GGTGTGAAACTGTCGTGCTCTGTT	TTCGAGAACTACCACGTCCATCCT	Mapping
Xtxp285	ATTTGATTCTTCTTGCTTTGCCTTGT	TTGTCATTTCCCCCTTCTTTCTTTT	Mapping

Name	Forward primer (5' to 3')	Reverse primer (5' to 3')	Purpose
SB2236	CCGTTTCTCAGCTTCTGTGTAGCA	GGACGTCTTCATGAACGTCAAATG	Mapping
Xtxp70	AGTGACCTTAGCACCAAGCTC	TCAGGTAGCACTAGAG	Mapping
Xtxp69	ACACGCATGGTTTGACTG	TTGATAATCTGACGCAACTG	Mapping
SB2343	GGATCGGAGTGATCCGTAGATAGC	CACAGTACACAACATGCCCATCAA	Mapping
SB2405	GTATTGTTATTCGGTGGAGGACGG	TATAGTGGATGGATGGATGAAGCG	Mapping
Xcup48	TCACTAGCGCCTCCAAAATC	TCCAATCCTTCCTGTGCTTC	Mapping
SB2435	TAGCTAGCTGCGTAATGACGAGGA	TCGAGCATCGTCGGAAAATATACA	Mapping
Xcup05	GGAAGGTTTGCAAGAACAGG	CCAGCCCAACAAGTGCTATC	Mapping
SB2485	ATCTCTCGCTGTGTCTCCTGTCCT	CACCTTCCTCTTGAACTCCGTCAC	Mapping
SB2550	GATTTCAGTCCCCGTTTGTTTTTG	GAATCATCTCACCACAGCATCACC	Mapping
SB2599	ATCTGGAGTCTGGAAGAATGCTGG	TGTTTCCACTAGTGCATCAGCACA	Mapping
Xtxp343	CGATTGGACATAAGTGTTC	TATAAACATCAGCAGAGGTG	Mapping
SB2647	GCTTGTGATCGAGTTGTGATGGAC	CTGCTGAACCAGGAGAACCAGG	Mapping
Xtxp24	TTGTGTAGTCCATCCGATGC	TTCTAAGCCCACCGAAGTTG	Mapping
Xtxp41	TCTGGCCATGACTTATCAC	AAATGGCGTAGACTCCCTTG	Mapping
SB2831	TTCAGCTTTGGATCTCGATCTGTG	CGTCAAATTCTAGCTCCTATCATCA	Mapping
SB2836	CTACATGCACTGATGCACCTCTCA	GACCGTCACTCGAATGTTCTCTGA	Mapping
Xtxp21	GAGCTGCCATAGATTTGGTCG	ACCTCGTCCCACCTTTGTTG	Mapping
Xtxp94	TTTCACAGTCTGCTCTCTG	AGGAGAGTTGTTCGTTA	Mapping
SB3083	GGATGAGCAAGACCCAAAGAGAGA	GCAGCAACTTCAGCACAAGGTATG	Mapping
Xtxp303	AATGAGGAAAATATGAAACAAGTACCAA	AATAACAAGCGCAACTATATGAACAATAAA	Mapping
SB3129	TCTCAGATGCGGTTGGTTACAAAA	CGCGTCACTCAAGCACATACTTCT	Mapping
SB3171	AATCGATCCCCAGAGTTTTGGAGT	GAAAAAGCAGCGTCACC7CATC	Mapping
SB3178	TTCTCTGCTCCGTTTCCTAACAGG	TCTCGATTCATCTCATCGTCTTGG	Mapping
Xtxp15	CACAAACACTAGTGCCTTATC	CATAGACACCTAGGCCATC	Mapping
SB3216	CTGTATGCATGTTTGCTACGAGGG	TCAAGTTCAAGCAAGCCACCATTA	Mapping
SB3221	GCAGGTACAAAAATACAACTTGGCTTG	CTATTCGCTTCAGCTTATCACCCG	Mapping
SB3233	GTGCTCACCCATATGACGAACCT	CATCTACAACTACTGCACCGACCG	Mapping
SB3271	ATCGATCACCAATCGAGCTAGAGG	TCGAGGTAAATGA7GCGTCTACA	Mapping
SB3317	TAGTAGTCGTAGGAGGTGGGTGGC	GGTTGTTAGGATTCCGCTATAAAGCCT	Mapping
SB3381	CCCTACATAAATAATGGCAGACCGA	GAGGAGGAGGAGCATCAATCACAT	Mapping
SB3385	TGCACCTTTAGTTACACCTTGTTTACC	AATCAACCTCCATCAACTATGCTG	Mapping
SB3411	GCTCGCATACTAGCTCATCGTTCA	GACTGTCCATGCGATCAGTAGGAA	Mapping
Xtxp6	ATCGGATCCGTCAGATC	TCTAGGGAGGTTGCCAC	Mapping
Xtxp145	GTTCCTCCTGCCATTACT	CTTCCGCACATCCAC	Mapping
SB3580	ATCTCGCAGTAATGGAACCCACAT	AGCTAGTCAAAGCAGGATCGATGG	Mapping
SB3233	GTGCTCACCCATATGACGAACCT	CATCTACAACTACTGCACCGACCG	Mapping
Xtxp274	GAAATTACAATGCTACCCCTAAAAGT	ACTCTACTCCTTCCGTCCACAT	Mapping
SB3613	CTTGTCTTCTGAAACGGAGGGAGA	GTCCATCTGACCGTTTCATCACAG	Mapping
SB3621	GG/TCTTGGCATTACCTGCAAAAG	GCAATTTGCACTGGTTACTTGCTG	Mapping
SB3631	AAAGCCAATCCGGGGAGATGT	CAATCATGGGATCGTACAAGGAGG	Mapping
SB3680	GTTGCGTGCTAATTTCTTTGCTCC	TAACCAAAAGCTAGCGCGAAGTG	Mapping
SB3705	AGTCGTTCGACCTGAGCCTTTCT	TATATCCATCGCTTTGGCGTCAT	Mapping
Xtxp95	TCTCCGTTTGCCCGCCAG	CACCGTACCGCCTCCCGAATC	Mapping
SB3772	ATTCCTCAAGGCTCTTTCGTGGAT	GACCGGTAAAAAGGAGATGCCATT	Mapping
SB3870	TAATAATGCTGCATGCTGAGTGCC	TCCATTGTGTTTCTGATAGCTTGACAG	Mapping
Xtxp159	ACCCAAAGCCCAAATCAG	GGGGGAGAAACGGTGAG	Mapping
SB3974	TGAACTGAAACCGCTGTCACCTAC	CAGTGCTATATCCGTGCTGCATTC	Mapping
SB3991	AAGGTCAGTGTCCATCTAGGCAAA	AGGGAAGTGTCAGGTCATAGTGCC	Mapping
SB3996	GAGCTTGTCGAGGAGGTAGTCCAG	TGTCCCCTATTTATACCCCCATCC	Mapping
SB4016	GACAAGAGAAATCCCGTAAACCCC	GTACCTCAACCAGACGCTCGTCAC	Mapping
SB4085	GTTATTCCCTGATGGATGGATGGA	GTCCACAAAGCAAAGAATCCCGTA	Mapping
SB4117	GGTGCAGAAATTATAAGGGAATTGG	AACCGAACGGAACAGAACAGCTT	Mapping

Name	Forward primer (5' to 3')	Reverse primer (5' to 3')	Purpose
SB4124	ACTACACCTCACTTAGGGCACCCA	GCTTTAAGTCACCCTCTTCAATAGTGC	Mapping
SB4143	TCTCCTTTAATTACGACCGACCGA	CTCCATGGATCGACCCTTTTGTT	Mapping
SB4168	CTCTGCTGGTTGGTGCTATTTCCT	CGGTAATAAAGGTGTCTCCAATGGT	Mapping
SB4174	GGAGGGGAGGTTGGTCACTAGACT	GGCCTTGGCTTCCTCCAGTAGTAT	Mapping
SB4182	ATCTTTCGCAGCTTAATGTGGAGC	CATGCTGCTGATGTCGCACTC	Mapping
SB4216	CACTCTCTACCACCACTGGCCTTT	AGATTTGGACTTGTGCCACTCCTC	Mapping
SB4217	TGGGAGAGGAGAATCTTCCATTCA	CAGGAGGAGGAATGTAAGGCATGT	Mapping
SB4259	CGACTGCTGCTGCTTTCTCAATAA	GATGCGTGTTTAGTGCAAAGATCG	Mapping
SB4267	AGCGGGTGAATACGAATACGAGAA	CTGCTCGTCCTCAACATCAGCTT	Mapping
SB4288	TCACAGCTTTGAACTGTGTGTTGC	CAAAACTTGTCCAAGCCATCACAT	Mapping
SB4386	TTCGTTTGCTACCTCACACAGCTT	CTCTGGTGACGATGGTTTTGATTG	Mapping
SB4391	GCATTAGACTACCCTTTGATGGCA	GAACTTAGGGGGAGCTGTTCGATT	Mapping
Xcup47	TGAGCAATGAACTTAGGGGG	CTACCCT7GATGGCAGTACC	Mapping
SB4419	ATATACCCCTTCTTCCCAGCTTGC	TGCATGCATGTTCTTCTCAGCATA	Mapping
SB4460	GCAATCAAGGATCCAGAGACCATT	TGCTTTTATTTGCCGTAGAATGCC	Mapping
SB4528	GTGCTACAGTGCATGGTTTTCCAA	CCCTCATTCTTCCCAACCTTCTCT	Mapping
SB4533	AAGGAGAGAGAGAGGGGGCAAAAG	CACGATCGTCTCTCTGGCTGTCTA	Mapping
Xtxp321	TAACCCAAGCCTGAGCATAAGA	CCCATTCACACATGAGACGAG	Mapping
Xtxp18	GCCTCTACATATCCACAAGCC	CAGATCAGTCATGCCACCTG	Mapping
SB4563	ACCATGGCAAAGGTCCTTGACTTA	TCCCATTCTTCAAGGAAATCATCG	Mapping
SB4654	CTGAAAACTGTTTGCCTGTGATCG	CTCGATTAGCTCAGCAACTCATGC	Mapping
SB4660	AACCACAACCACGGGATATCGTC	ACGAAGAACGGATGCTCGTACTG	Mapping
Xtxp289	AAGTGGGGTGAAGAGATA	CTGCCTTTCCGACTC	Mapping
SB4706	ATTGCTTGCTCCTCCTGGGACT	GTTGCCTTTGCCTAGCTTTCCTTT	Mapping
SB4715	AATTCGAGAAGAGCTGGTGAGTGG	GTTTAGTGCGCGTGAGTAGTGCAT	Mapping
SB4725	AACCCCACCGTAACATCAGTCAGT	AATGGCAGCTACCACTCACTCCTC	Mapping
SB4746	CGTGATGACAAAAACGTCTTCCAC	TGATCGCAGCACTGTTCATCATTA	Mapping
SB4751	GTAGACGATAGTCTGAAGCCATCTG	ACGACGCCGTGCACTTTAATTACT	Mapping
SB4759	TTCCTTCTTCTCTGTCGCTGGCTA	CTACCGACGAATCTCCGCAAAAC	Mapping
SB4780	TTGAGTTGCTTCTAAATCGCCCAT	TGACGGCCCTGTTCATAAAGAAAT	Mapping
Xtxp10	ATACTATCAAGAGGGGGAGC	AGTACTAGCCACACGTCAC	Mapping
SB4925	CCTGTGCTACCAGCAGAGATCGAG	TAGATATAGATGGCCGCCTCGTCA	Mapping
SB4951	GTGCCTTGTTTGCTATTCAAGCCT	CACAGTTTGGTAATGGAGCAACCA	Mapping
SB4958	ACGCAACTAGAATCGAGATAGCCG	GTGCCGTCCGGCATCAAGAAA	Mapping
SB5007	GGAACTTGTTGCTGGTGTAGCAGA	GCTGCCTCTTCGTACTGTGAGTGA	Mapping
Sb4-32	CTCGGCGGTTAGCACAGTCAC	GCCCATAGACAGACAGCAAAGCC	Mapping
SB5042	ACAGTAACCACCACCAGAACGCTT	TTCGCTTCTCTTCCCTTGTGTCTT	Mapping
SB5045	CACGTGGTCAGAATCACGAGAAGT	GACCTCAGGGTAATCTTCCTCGGT	Mapping
SB5058	GAGAATTGGAAGAAAGCCTCGGTT	CAGAGCTCCTAAACGGTCCTCAAA	Mapping
Xtxp107	CAAAGTGAGCGTGGTC	GGACAGGGATAACATAACATA	Mapping
SB5077	CATTCTTTGGTCACTTGGGGATTC	AAAAGGGTCTTCATTCACCAAGGC	Mapping
SB5095	CCTTCGCTCTCACTTCTTCCTCTG	GTGGAGTATGTAGTATGCGCGGTG	Mapping
SB5111	AAACCAACCCGACCCACTCTTTAT	CGGTTCGTTCCATCAGATTCTTCT	Mapping
Sb5-206	ATTCATCATCCTCATCCTCGTAGAA	AAAAACCAACCCGACCCACTC	Mapping
SB5144	TCGTTGTTCTACCCTGTCTCCCTC	CACTGGAAGAGATAACCGTGCTGA	Mapping
SB5155	GAAAAAGGAGCATCATCAGGCAGT	GGTGCTCTGGCGACTCATCAT	Mapping
SB5159	TATGCTTCCAATACAGAGGGACCG	TGTACGCCAGGAGATGTGATATGG	Mapping
SB5232	ATTGAAACAGAGCATCCTGCACAT	TGAATGATGCTTGCGTGTGTATTG	Mapping
SB5293	TGAATAATGCACGCAGTAGCGTCT	TATTCCCACGGCTCGCTAGCTACT	Mapping
SB5388	AGCGCAGAGAATCCAAATCTATCG	GTC7CTCCGTTTGTTCAT7CGC	Mapping
SB5393	CATCGAAACGGAAGACACAAAACA	CCTACAGTGTGTCTGGCTGCAAGT	Mapping
SB5407	GAGTCTGCGCTTATTGTGCCTTTT	TGCCTTTTTGCCAGATCTTTCTTC	Mapping
SB5520	CGCTCCTGTATCTGAAGAGGGAAA	TTCTCGGGAATTCATCCGCTACTA	Mapping

Name	Forward primer (5' to 3')	Reverse primer (5' to 3')	Purpose
SB5556	AGCTTCAAAGATGGCCAAAACAGA	GCAGAGCCACAAGTCAAGGTG7A	Mapping
SB5569	GCATCATCTTCGATTCCAGCAGAT	GCCTTCTTGAGCTTGAGGATATGG	Mapping
Xcup07	CTAGAGGATTGCTGGAAGCG	CTGCTCTGCTTGTCGTTGAG	Mapping
Sobic.007G204600_1	CACCCTCTCGCTAACGACAC	CTGTATCCGTAGAGCATGTC	Sequencing
Sobic.007G204600_2	CATAGCCTATTCCGATGCTG	CGTTGCTTGAATCATGCTTG	Sequencing
Sobic.007G204600_3	TAACTGGCAAGGCGACCTAC	TCCAGAGTCTCTTATGCCAC	Sequencing
Sobic.007G204600_4	ATTGCCCATCCTTGTGTCAG	TACCCAGTATCTGCAACATC	Sequencing
Sobic.007G204600_5	ATAGATAGCAATGCTGATGC	GGGTAGCATAGAAGCACTCC	Sequencing
Sobic.007G204600_6	TGGAACGTTTGGTCAGAGTG	ACCAGCAAATCCATCTGTGC	Sequencing
Sobic.007G204600_7	CCTTCGGCTAAATCGGCATC	TCCCCACAATTCCAGCTAAG	Sequencing
Sobic.007G204600_8	TTTGTATGTGCACACGATGG	TCACCCATGTAGAACATTGG	Sequencing
Sobic.007G204600_9	TAAAGTGGAGCTGAAGTCAC	CCACTGGCTCCCATCGGAAC	Sequencing
Sobic.007G204600_10	CCAAAGGCGAGATCTACGTG	AGGAAGTTCTTGCAACAGTC	Sequencing
SbSu_qRT	ACGCTAAGGCAGTGGTAAGC	AGGTCGCCTTGCCAGTTAAA	qRT-PCR
SbIsa2_qRT	ATTGCAACCATCCGGTGACT	AGAAGTGGAGGCCGTGAAAG	qRT-PCR
SbIsa3_qRT	GCAGTCCTCTTGATGCACCT	TTCCATTCAGCCCACCTGTC	qRT-PCR
SbPul1_qRT	GCACTGGCATTGGTAGTTTCA	TGCGCCTTGTCTCTGTTTCA	qRT-PCR
SbSbeI_qRT	CTCTGAGTGGTCGATGGGTG	TGTCGCCAACGATAGACTGA	qRT-PCR
SbSbeIIa_qRT	ATTCACCGGGAACTATGGCG	GGCATTCCGCTGACATCTTC	qRT-PCR
SbSbeIIb_qRT	AGCACTTCACTACCGACTGTT	GCTATCATTCCGCTGGAGCA	qRT-PCR
SbSbeIII_qRT	GGATTGTAGCAACCTTGCAGTTAT	AATGAGGAGCGCATGTGGAT	qRT-PCR
SbSe1_qRT	GAGGGCAGATGTGGATGAGA	CACGAAAACTCTGTCGTCGTC	qRT-PCR
SbSh1_qRT	ACTGGTGGCCAGGTTGTGTA	TAGTCCCAACAGCATCAGGC	qRT-PCR
SbBt1_qRT	AGCAAGGCCATCGAGCATTT	GGGATAGGTGCACAAGGTCG	qRT-PCR
SbSh2_qRT	TGTTGATGAGAGCCGAGCTT	CTCTGCAACATCAGTAGCATCG	qRT-PCR
SbSsI_qRT	GAGAACAGGGTACAGGGTGG	CTTCATGAGCCCTTCCCAGG	qRT-PCR
SbPho1_qRT	CGTACCGTGATCAGAAATTATGGAC	CCTGATATCCACCTGTCTAGGG	qRT-PCR
SbSweet1-2_qRT	CCAGGGAGACCAAGGCGAA	TGCCGCGTACATCCCTATCG	qRT-PCR
SbSweet4-3_qRT	ACCTAGAGCAGCAAGAGGGA	GTAGAACGTCGGCAGTGGC	qRT-PCR
SbUbi_qRT	GGTTCGGGAGGTGGCCTAGGT	AGCATGTACATTCCCAGCGGTA	qRT-PCR
